# Activity-Regulated Cytoskeleton-Associated Protein (Arc/Arg3.1) is Transiently Expressed after Heat Shock Stress and Suppresses Heat Shock Factor 1

**DOI:** 10.1038/s41598-019-39292-1

**Published:** 2019-02-22

**Authors:** A Young Park, Yeon Seung Park, Dami So, In-Kang Song, Jung-Eun Choi, Hee-Jung Kim, Kong-Joo Lee

**Affiliations:** 1 College of Pharmacy and Graduate School of Pharmaceutical Sciences, Ewha Womans University, Seoul, 03760 Korea; 2 Present Address: Spark biopharma, #203-207A, Seoul National University, 1 Gwanak-ro, Gwanak-gu, Seoul, 08826 Korea

## Abstract

Heat shock proteins are induced by activation of heat shock factor 1 (HSF1) in response to heat shock and protect against heat stress. However, the molecular mechanisms underlying the downstream signal of heat shock have not been fully elucidated. We found that similarly to canonical Hsps, Arc/Arg3.1 is also markedly induced by heat shock and by other cellular stress inducers, including diamide, sodium arsenite and H_2_O_2_ in various cells. We noted that heat stress–induced Arc/Arg3.1 protein is short lived, with a half-life of <30 min, and is readily degraded by the ubiquitin–proteasome system. Arc/Arg3.1 overexpression inhibited the up-regulation of heat shock–induced Hsp70 and Hsp27, suggesting that Arc/Arg3.1 is a negative regulator of heat shock response (HSR). Studying the effect of Arc/Arg3.1 on HSF1, a major transcription factor in HSR, we found that Arc/Arg3.1 binds to HSF1 and inhibits its binding to the heat shock element in gene promoters, resulting in reduced induction of Hsp27 and Hsp70 mRNAs, without affecting HSF1′s phosphorylation-dependent activation, or nuclear localization. Arc/Arg3.1 overexpression decreased cell survival in response to heat shock. We conclude that Arc/Arg3.1 is transiently expressed after heat shock and negatively regulates HSF1 in the feedback loop of HSR.

## Introduction

When cells are exposed to environmental stresses including heat shock, oxidative stress, hypoxia, and toxic chemicals such as sodium arsenite, diamide, and amino acid analogues, the cellular defense system called heat shock response (HSR), is provoked^1,2^. A major feature of HSR is the induction of heat shock proteins (Hsps). Upon activation by various stresses, heat shock factor 1 (HSF1) is phosphorylated, forms trimers and is translocated to the nucleus^3^. In the nucleus, the activated trimeric HSF1 binds to heat shock element (HSE) and initiates the transcription of *hsp* genes. Active HSF1 trimers are inactivated by interacting with Hsp70 and Hsp40^4^, or with hnRNP K^5^, which inhibit its DNA binding capacity, resulting in reduced transcription of the *hsp* genes. Inactivation of HSF1 also occurs due to post-translational modifications such as acetylation, sumoylation or phosphorylations^6^. Another feature of HSR is the induction of thermotolerance in the cells primed with mild stress, which makes the cells resist the lethal stresses including heat shock, oxidative stress, sodium arsenite and diamide etc^2,7–10^. It is known that the chaperonic functions of Hsps are connected to the induction of thermotolerance, because Hsps can repair and remove the misfolded and denatured proteins and maintain cellular protein homeostasis^11^. Phosphoproteomics^12^ and microarray analysis^13^ helped comprehensive understanding of HSR.

As part of our ongoing studies of heat shock response, we conducted microarray studies of radiation induced mouse fibrosarcoma cell line, RIF-1, and its thermotolerant variant, TR-RIF-1 (TR). Among the 12,339 genes revealed in the microarray studies, 2,208 were up- or down-regulated more than 2 fold with *p*-values < 0.05^13^. Arc/Arg3.1 (Activity-regulated cytoskeleton-associated protein) was the most upregulated entity. Arc/Arg3.1 is a 45.3 kDa (396 amino acids) protein, rich in proline, glutamine, arginine and glutamic acid residues. Arc/Arg3.1 in brain has been well studied. It is an immediate-early gene (IEG), which is expressed at low levels under resting condition, but its transcription is rapidly and transiently induced in response to external stimuli and intense synaptic activity^14,15^. *Arc* gene expression can be induced by various stimuli in brain including in hippocampus and cortex following seizure-inducing activity, BDNF, activation of mGluR, growth factor stimulations including NGF, EGF and PDGF^16^ and sleep-waking cycle^17^. Arc/Arg3.1 is an attractive marker of neuronal activity, because Arc/Arg3.1 plays key roles in multiple forms of learning and memory by regulating seemingly opposing forms of neuronal plasticity; long-term potentiation (LTP) and long-term depression (LTD), and homeostatic plasticity^18^.

Molecular function of Arc/Arg3.1 has been attributed to interactions with dynamin, a large GTPase essential for intracellular membrane trafficking including clathrin-mediated synaptic vesicle recycling, and endophilin, a protein playing a role in vesicle formation and function. These interactions enhance the endocytosis of AMPA receptors which contributes to the synaptic transmission in LTD and homeostatic plasticity reduction^19^. In early phase of LTP, sustained Arc/Arg3.1 synthesis is required to generate stably modified synapses by expanding the actin cytoskeleton^20^. In late phase of LTP, Arc/Arg3.1 promotes endocytosis of the AMPA receptors in inactivated post synapses that previously experienced strong activation^21^. The induction of Arc/Arg3.1 highly correlates with augmented neuronal activity that is required for cognitive processes such as learning and memory consolidation^22^. Recently, Arc/Arg3.1 function in schizophrenia was reported^23^. The retroviral/retrotransposon GAG-like domain in Arc/Arg3.1 forms virus-like capsids and transports self mRNA in neuronal cells^24,25^. Most physiological studies on Arc/Arg3.1 have been performed in the neuronal system and its role in other systems is poorly understood.

Although HSR, a cellular defense mechanism against various stresses, has been extensively characterized regarding activation of heat shock gene transcription by HSF1 and drastic repression of normal protein synthesis pathways, the molecular mechanisms underlying the stress and down-stream signal transduction of heat shock still need to be clarified. We firstly found the significant induction of Arc/Arg3.1 in non-neuronal system in response to heat shock and related stresses. We demonstrated that the induction kinetics of Arc/Arg3.1 in response to heat shock are similar to those of Hsps, except for its faster response and shorter lifetime. In our HSR system, we found that Arc/Arg3.1 inhibits the induction of Hsp27 and Hsp70 both in mRNA and protein levels by inhibiting the function of heat shock-induced HSF1 by reducing its binding to HSE. These studies reveal a novel function of Arc/Arg3.1 in HSR of non-neuronal system.

## Results

### Arc/Arg3.1 gene is markedly up-regulated in response to heat shock

**Figure 1:**
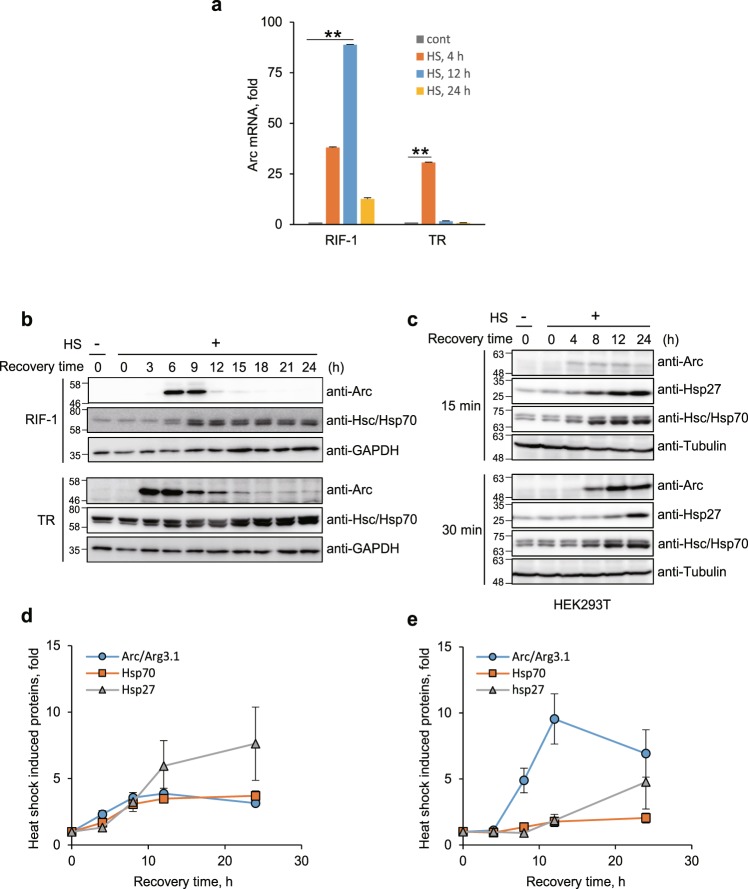
Arc/Arg3.1 is induced by heat shock. (**a**) RIF-1 cells were exposed to heat shock at 45°C for 30 min and recovered from the stress in fresh media at 37°C for 4, 12 and 24 h. Each sample was analyzed by Illumina microarray analysis. mRNA levels of the most up-regulated gene, *Arc/Arg3.1*, are shown in bar graph. Data were presented as the means ± S.D. of duplicated experiments (t-test; *P < 0.05, **P < 0.01). (**b)** RIF-1 and TR cells were heat shock treated at 45°C for 25 min and recovered for the indicated times in fresh media. Arc/Arg3.1 and Hsc/Hsp70 were analyzed by Western analysis with their specific antibodies. As a loading control, GAPDH level was detected using anti-GAPDH antibody. Western blot results were selected representative data from triplicated results. (**c**) HEK293T cells were exposed to heat shock at 45°C for 15 and 30 min and recovered for the indicated times. Arc/Arg3.1, Hsp27 and Hsc/Hsp70 were detected by Western blot analysis with their specific antibodies. As a loading control, tubulin level was detected using anti-tubulin antibody. Western blot results were selected representative data from triplicated results. Quantified results of triplicated Western images were shown in (**d**,**e**) for 15 min and 30 min heat shock treatment, respectively. Data were presented as the means ± S.D.

To investigate the comprehensive microarray study in response to heat shock, we examined the mRNA expression profiles employed radiation induced mouse fibrosarcoma cell line, RIF-1, because this has its thermotolerant variant cell line, TR-RIF-1 (TR)^13^. RIF-1 and TR cells were exposed to heat shock (45°C for 30 min) and recovered from the stress in fresh media at 37°C for 4, 12 and 24 h and their mRNA profiles were analyzed by microarray study. Among identified 12,339 genes in microarray assays, 2,208 were up- or down-regulated more than 2 fold with *p*-value < 0.05^13^. *Arc/Arg3.1* was the most up-regulated gene and *Arc/Arg3.1* mRNA was induced 38-, 89- and 13- fold in RIF-1 cells at 4, 12 and 24 h recovery after heat shock, and 31-, 2- and 1- fold in TR cells (Fig. 1a). In addition to *Arc/Arg3.1*, immediate early genes (IEGs) including *Egr1, Fos, Fosb, Jun, Junb* were also induced (Supplementary Fig. 1a). We confirmed the protein levels by Western analysis using anti-Arc antibody. Arc/Arg3.1 was almost undetectable in control cells, but transiently appeared during recovery after heat shock, and again disappeared. Since Arc/Arg3.1 mRNA and protein were up- and down-regulated during recovery after heat shock, we investigated the kinetics of the changes in its expression during recovery after heat shock by Western analysis using anti-Arc antibody. RIF-1 and TR cells were exposed to heat shock at 45°C for 25 min, and recovered for the indicated times at 37°C. The endogenous expression of Arc/Arg3.1 was negligible in control cells, and was induced at 6 h recovery after heat shock and remained up to 12 h in RIF-1 cells. In thermotolerant TR cells, Arc/Arg3.1 was induced in 3 h after heat shock, faster than that in RIF-1 cells (Fig. 1b). Since the induction of Hsps is the major feature of HSR, Hsp70 induction was monitored employing Western analysis with anti-Hsc/Hsp70 antibody. As shown in Fig. 1b, the induction of Arc/Arg3.1 in response to heat shock was similar to that of Hsp70, during recovery after heat shock. These results indicate that Arc/Arg3.1 is induced by heat shock similarly to Hsp70, but the induction of Arc/Arg3.1 was transient, compared to that of Hsp70. Earlier induction of Arc/Arg3.1 in TR cells suggests that Arc/Arg3.1 is related to the generation of thermal resistance of cells and that it plays a key role in HSR signaling pathways.

### Induction of Arc/Arg3.1 depends on the degree of heat shock stress

Heat shock causes immediate inhibition of cellular protein synthesis, which was recovered by synthesis of Hsps followed by gradual synthesis of other proteins^26^. The kinetics of protein synthesis and HSR signaling vary depending on the amounts of stress. To investigate whether the induction of Arc/Arg3.1 correlates with the degree of stress, we examined the expression of Arc/Arg3.1 after treating cells with different amounts of heat shock stress. HEK293T cells were exposed to 45°C for 15 and 30 min and recovered for the indicated times, and Arc/Arg3.1, Hsp27 and Hsp70 expressions were measured by Western analysis (Fig. 1c–e). At mild heat shock (at 45°C for 15 min), the extent of Arc/Arg3.1 induction was low. At moderate heat shock stress (at 45°C for 30 min), with presumed 50% cell survival after stress, Arc/Arg3.1 expression was considerably higher, and reached maximum at 12 h recovery, the kinetics of Hsp70 induction was similar to that of Arc/Arg3.1 induction. Arc/Arg3.1 expression in response to heat shock were confirmed with two kinds of antibodies against Arc (Santa Cruz, anti-Arc E-7 and C-7) as shown in Supplementary Fig. 2a. Hsp27 was induced later than Arc/Arg3.1. These results indicate that the expression of Arc/Arg3.1 correlates with the amount of heat shock stress.

### Arc/Arg3.1 is induced in response to various chaperone inducing stresses

It is known that cells primed with mild heat shock or other stresses (e.g. sodium arsenite, diamide, ethanol, H_2_O_2_) acquire a transient resistance from elevated levels of inducible Hsps^2,7,13^. We investigated possible similarities between Arc/Arg3.1 and other chaperone Hsps induced in response to various stresses, by examining Arc/Arg3.1 expression in response to various Hsp inducing stresses.

**Figure 2:**
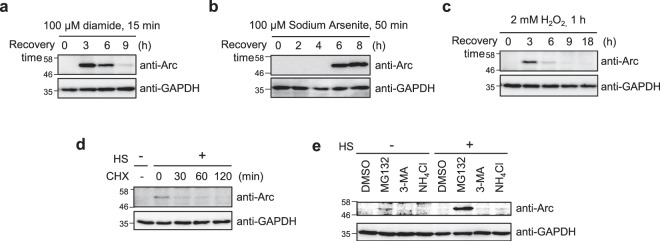
Arc/Arg3.1 is induced by various stresses and degraded in proteasome. (**a**–**c**) HeLa cells were treated with 100 μM diamide for 15 min (**a**), 100 μM sodium arsenite for 50 min (**b**) or 2 mM H_2_O_2_ for 1 h (**c**). Cells were recovered for the indicated times with fresh media. Arc/Arg3.1 was analyzed by Western analysis using anti-Arc/Arg3.1 antibody. As loading controls, GAPDH and tubulin levels were detected using anti-GAPDH and anti-tubulin antibody. (**d**) HeLa cells were heat shock treated at 45°C for 25 min and recovered for 9 h in fresh media at 37°C to induce maximum Arc/Arg3.1 expression. Then cells were treated with 10 μg/mL cycloheximide (CHX) for the indicated times. The endogenous Arc/Arg3.1 was detected by Western analysis using anti-Arc antibody. As a loading control, GAPDH level was detected using anti-GAPDH antibody. (**e**) After heat shock stress at 45°C for 25 min, HeLa cells were recovered for 9 h in fresh media. Then cells were incubated with 10 μM MG132, 10 mM 3-methyladenine (3-MA) or 10 mM NH_4_Cl for 6 h. The cell were analyzed by Western analysis using anti-Arc/Arg3.1 antibody. As a loading control, GAPDH level was detected using anti-GAPDH antibody. Western blot results were selected representative data from more than triplicated results.

Diamide, a disulfide crosslinker, and sodium arsenite, a labeling reagent of cysteine residues, which cause protein misfolding, induced the Hsps^2^. HeLa cells were exposed to various stresses of equivalent degree (100 μM diamide for 15 min, 100 μM sodium arsenite for 50 min, 2 mM H_2_O_2_ for 1 h), recovered with fresh media for the indicated times (Fig. 2a–c). MG132 proteasome inhibitor (50 μM MG132 for 1 h and recovered in various times) induced Hsps^13^, but not much induced Arc/Arg3.1 in RIF-1 cells (Supplementary Fig. 1a,b). Induction of Arc/Arg3.1 was observed during recovery after all of the stress treatments. Stresses that strongly induced Hsps (diamide and sodium arsenite)^2^ also strongly induced Arc/Arg3.1. In order to identify whether induction of Arc/Arg3.1 by heat shock is occurred in neuronal cells, we examined heat shock induced Arc/Arg3.1 in neuroblastoma SH-SY5Y cells. As shown in Supplementary Fig. 2b, SH-SY5Y showed similar kinetics of Arc/Arg3.1 induction as well as non-neuronal cells. These results indicate that Arc/Arg3.1 expression occurs in response to a variety of cellular stresses inducing protein misfoldings, like other Hsps, suggesting that Arc/Arg3.1 upregulation can be another novel universal marker of cellular stress like that of Hsps.

Since Arc/Arg3.1 expression in response to various stresses are similar to Hsps, we investigated whether Arc/Arg3.1 is a target gene of HSF1 by employing HSF1(−/−) mouse embryonic fibroblast (MEF) cells. MEF HSF1(+/+) and HSF1(−/−) cells were exposed to heat shock at 45°C for 20 min and recovered at 37°C for the indicated times. HSF1, Arc/Arg3.1, Hsp27 and Hsc/Hsp70 were detected by Western blot analysis with their specific antibodies. As shown Supplementary Fig. 3a, Arc/Arg3.1 expression was induced in HSF1(−/−) MEF cells in response to heat shock as well as in WT cells, while Hsp27 and inducible Hsp70 expression were not induced in HSF1(−/−) MEF cells. This was confirmed with bioinformatics analysis by comparing the sequence of heat shock element (HSE), HSF1 binding element, with that of Arc/Arg3.1 promoter from Eukaryotic Promoter Database (GeneCards and EPDnew). There is no similarity between HSE and Arc/Arg3.1 promoter sequence (Supplementary Fig. 3b). The results indicate that Arc/Arg3.1 is not a target gene of HSF1.

### Induced Arc/Arg3.1 is short lived and is readily degraded by ubiquitin-proteasome system (UPS)

Stress induced Arc/Arg3.1 was transiently expressed and degraded, unlike Hsp70 (Fig. 1b,c). We examined the degradation rate of Arc/Arg3.1 employing cycloheximide (CHX), an inhibitor of mRNA translation, with the goal of investigating its half-life. HeLa cells were exposed to heat shock at 45°C for 25 min and recovered at 37°C for 9 h to induce maximum level of Arc/Arg3.1, then treated with CHX for 0, 30, 60 and 120 min. As shown in Fig. 2d, Arc/Arg3.1 was induced by heat shock at 9 h recovery (lane 2) and was degraded to more than 50% in 30 min with CHX, but in several hours without CHX (Supplementary Fig. 4). Based on a regression analysis of Arc/Arg3.1 protein levels, the half-life of Arc/Arg3.1 protein appeared to be ~30 min. Since half-life of most proteins ranges between 45 min to 22.5 h in general^27^, the turnover of Arc/Arg3.1 seems to be more rapid than that of most proteins.

We attempted to determine the degradation pathway of Arc/Arg3.1. HeLa cells were exposed to heat shock at 45°C for 25 min and recovered at 37°C for 9 h with fresh media, in which maximum Arc/Arg3.1 was expressed. The cells were then treated with three types of degradation inhibitors: 10 μM MG132 (proteasomal degradation inhibitor), 10 mM NH_4_Cl (lysosomal degradation inhibitor) and 10 mM 3-methyladenine (3-MA, autophagosomal degradation inhibitor) for 6 h. Cellular proteins were separated on SDS-PAGE and detected with anti-Arc and anti-GAPDH antibodies (Fig. 2e). Arc/Arg3.1 accumulation was detected in cells treated with the proteasomal degradation inhibitor, MG132, even in control cells without heat shock treatment, compared to control DMSO sample (lane 2). Moreover, substantial amounts of Arc/Arg3.1 accumulation was detected in cells treated with MG132 after heat shock and recovery. There were no discernible changes in Arc/Arg3.1 in control cells treated with DMSO, lysosomal degradation inhibitor NH_4_Cl, or autophagosomal degradation inhibitor 3-MA. The results confirm that both control and stress-induced Arc/Arg3.1s have short half-lives as they are readily degraded in ubiquitin-proteasome system (UPS).

### Arc/Arg3.1 suppresses the expression of Hsps

**Figure 3:**
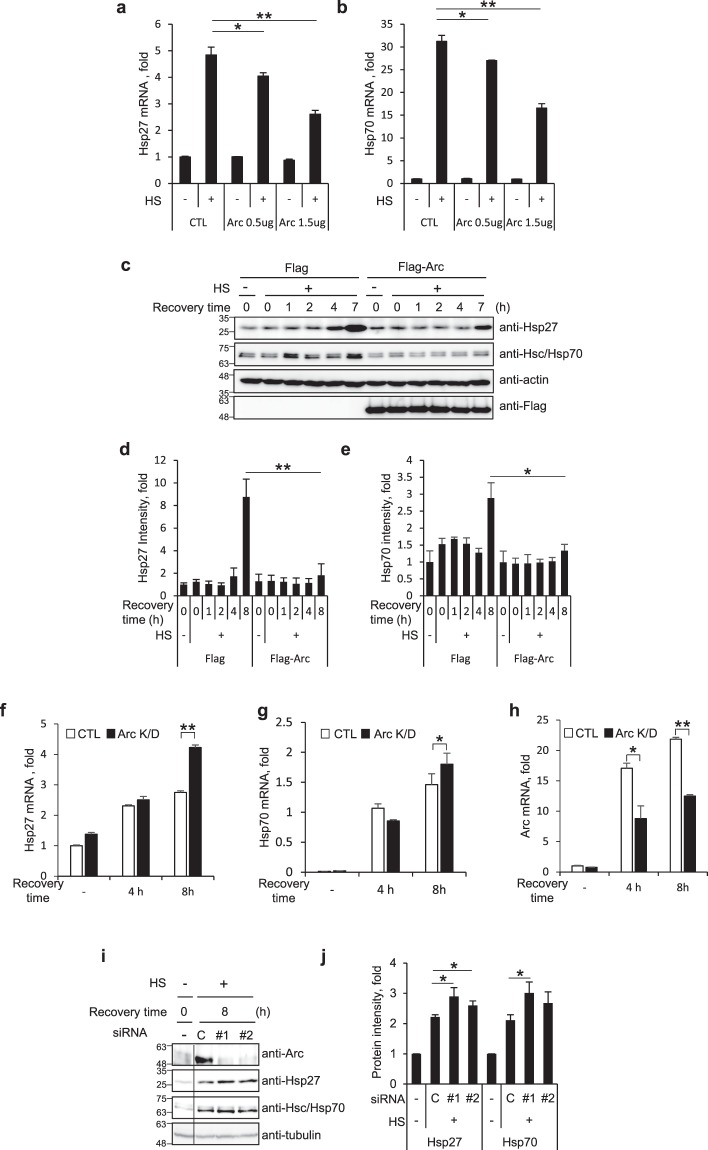
Arc/Arg3.1 inhibits Hsp27 and Hsp70 induction. (**a**,**b**) HEK293T cells were transfected with Flag empty vector or Flag-Arc. Cells were exposed to heat shock at 45°C for 15 min and recovered for 5 h. Quantitative RT-PCR was performed using primers for Hsp27 (**a**) and Hsp70 (**b**) mRNA. Relative quantities of Hsp27 mRNA and Hsp70 mRNA were normalized against GAPDH mRNA. (**c**–**e**) HEK293T cells overexpressing Flag or Flag-Arc/Arg3.1 were exposed to heat shock at 45°C for 15 min and recovered for the indicated times. Cells were analyzed by Western blot analysis using anti-Hsp27, anti-Hsp70, anti-β-actin and anti-Flag antibodies (**c**). Quantified amounts of Hsp27 (**d**) and Hsp70 (**e**) were represented. (**f**–**h**) HeLa cells were transfected with control siRNA and Arc/Arg3.1 siRNA #1. After 48 h, cells were exposed to heat shock at 45°C for 15 min, and recovered at 37°C for the indicated times. *HSP27* mRNA (**f**), *HSP 70* mRNA (**g**) and *Arc/Arg3.1* mRNA (**h**) were analyzed using quantitative RT-PCR and normalized to GAPDH mRNA. (**i**,**j**) HEK293T cells were transfected with control siRNA and Arc/Arg3.1 siRNAs (#1, #2), heat shock treated at 45°C for 15 min and recovered for 4 h or 8 h. Cells were analyzed by Western blot analysis using anti-Arc, anti-Hsp27, anti-Hsp70 and anti-tubulin antibodies (**i**). Quantified amounts of Hsp27 and Hsp70 in 8 h recovered cells were represented (**j**). Data were presented as the means ± S.D. of triplicated experiments (t-test; *P < 0.05, **P < 0.01). Western blot results were selected representative data from more than biologically triplicated results.

The most distinct feature of HSR is the activation of HSF1 and the resulting induction of mRNAs and proteins of Hsps. Arc/Arg3.1 induction pattern was similar to that of Hsps and dependent on the amount of heat shock. Hsp70 and Hsp40 have been reported to regulate HSR through binding to HSF1 resulting in inactivation of HSF1 as a negative feedback loop^3,4^. We questioned whether Arc/Arg3.1 also functions in the regulation of HSR as a new modulator. To investigate whether Arc/Arg3.1 expression affects the expression of Hsps, we examined mRNA and protein levels of heat shock induced Hsps in the presence and absence of Arc/Arg3.1. HEK293T cells transiently transfected with Flag empty vector, 0.5 μg or 1.5 μg of Flag-Arc were exposed to heat shock at 45°C for 15 min and recovered at 37°C for 5 h, and mRNA levels of *hsp*27 and *hsp*70 were assessed by quantitative RT-PCR. As shown in Fig. 3a,b, mRNA levels of *hsp*27 and *hsp*70 were increased by heat shock treatment, but decreased by Arc/Arg3.1 overexpression in a dose dependent manner. Overexpression of Arc/Arg3.1 by transfection of 1.5 μg of Flag-Arc reduced the heat shock induced mRNAs of *hsp*27 and *hsp*70 to about 50% suggesting that Arc/Arg3.1 inhibits the induction of stress-induced *hsp*27 and *hsp*70 genes.

To confirm the inhibitory effect of Arc/Arg3.1 on *hsp* mRNA induction, we examined the effect of Arc/Arg3.1 overexpression on induced Hsp protein levels. HEK293T cells were transiently transfected with Flag empty vector or Flag-Arc, exposed to heat shock at 45°C for 15 min and recovered at 37°C for the indicated times. Cells were analyzed by Western analysis (Fig. 3c) and quantified protein intensities were expressed by bar graphs (Fig. 3d,e). The expressions of Hsp27 and Hsp70 were significantly reduced when Arc/Arg3.1 was overexpressed, compared to control empty vector transfected cells. The results indicate that Arc/Arg3.1 overexpression inhibits the induction of Hsps.

This inhibition effect of Arc/Arg3.1 was confirmed in studies employing Arc/Arg3.1 depleted cells. Arc/Arg3.1 siRNA (#1) was used for Arc/Arg3.1 knock-down in HeLa cells. The cells were exposed to heat shock at 45°C for 15 min and recovered for 4 and 8 h, and the mRNAs of *hsp*27 and *hsp70* were measured by quantitative RT-PCR. As shown in Fig. 3f,g, mRNA levels of *hsp*27 and *hsp70* increased to the same level in both control siRNA and Arc/Arg3.1 siRNA transfected cells in 4 h recovered cells, but were different in 8 h recovered cells. It was under about 50% knock down of Arc/Arg3.1 condition (Fig. 3h). We also tested Arc/Arg3.1 depletion effect on protein levels of Hsp27 and Hsp70. We transfected HEK293T cells with two different *Arc/Arg3.1* targeting siRNAs (#1 and #2). The cells were then exposed to heat shock at 45°C for 15 min and recovered for 8 h, and Arc/Arg3.1, Hsp27 and Hsc70/Hsp70 were detected by Western analysis. As shown in Fig. 3i,j, heat shock induced Hsp27 and Hsp70 levels were increased while heat shock induced Arc/Arg3.1 induction was inhibited by Arc/Arg3.1 siRNAs (#1, #2). These studies on the effects of overexpression and knock-down of Arc/Arg3.1 indicate that Arc/Arg3.1 inhibits heat shock induced induction of *hsp* genes.

### Arc/Arg3.1 inhibits HSF1 activation by preventing the binding of HSF1 to heat shock element (HSE)

**Figure 4:**
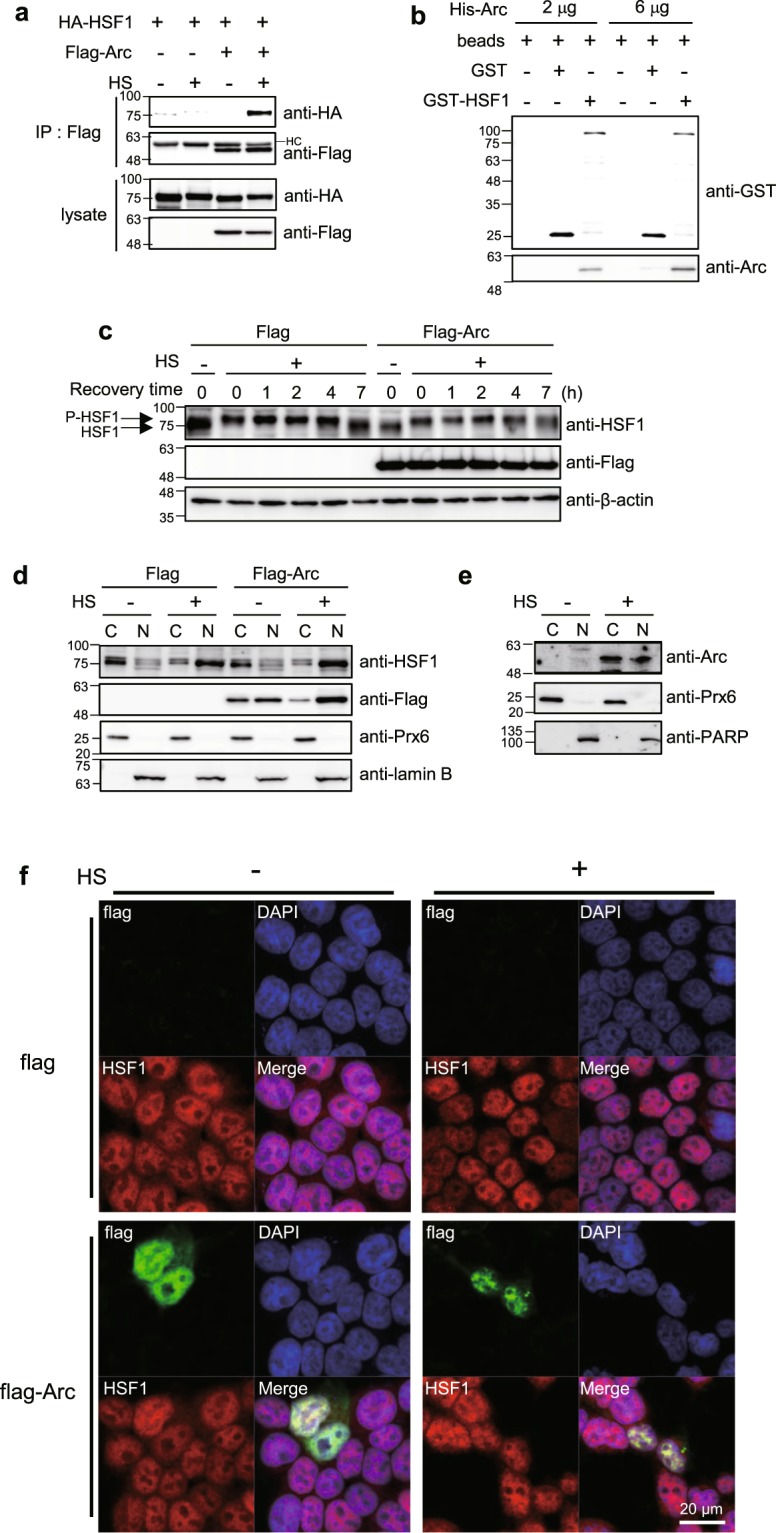
Arc/Arg3.1 binds with HSF1. (**a**) HEK293T cells were transfected with HA-HSF1 and Flag empty vector or Flag-Arc. After exposure to heat shock at 45°C for 15 min, immunoprecipitation was performed using anti-Flag antibody. Protein complex was analyzed by Western blot analysis using anti-HA and anti-Flag antibodies. (**b**) GSH beads, beads bound GST and beads bound GST-HSF1 were incubated with purified His-Arc. After washing step, beads bound proteins were analyzed by Western analysis using anti-GST and anti-Arc antibodies. (**c**) HEK293T cells were transfected with Flag empty vector or Flag-Arc. Cells were exposed to heat shock at 45°C for 15 min and recovered for the indicated times. Cells were analyzed by Western analysis using anti-HSF1, anti-Flag and anti-β-actin antibodies. P-HSF1; phosphorylated HSF1. (**d**) HEK293T cells were transfected with Flag empty vector or Flag-Arc. Cells were exposed to heat shock at 45°C for 15 min and fractionated into cytosolic (C) and nuclear (N) fractions. Each fraction was analyzed by Westen analysis using anti-HSF1, anti-Flag, anti-Prx6 and anti-Lamin B antibodies. Prx6; the cytosol marker, lamin B; the nucleus marker. (**e**) Hela cells were heat shock treated at 45°C for 25 min and fractionated into cytosolic (C) and nuclear (N) fractions. Each fraction was analyzed by Western analysis using anti-Arc, anti-Prx6 and anti-PARP antibodies. Prx6; the cytosol marker, PARP; the nucleus marker. (**f**) HEK293T cells were plated on the glass coverslip 24 h before transfection. Cells were then transfected with Flag or Flag-Arc. After 24 h, cells were treated with heat shock at 45°C for 15 min and visualized Flag-Arc (green), HSF1 (red) and nucleus (blue) under confocal microscopy. All of the Western blot results were selected representative data from more than duplicated results.

To investigate the mechanism underlying the inhibition of Hsps expression by Arc/Arg3.1, we employed immunoprecipitation assays to determine whether Arc/Arg3.1 interacts with HSF1. HEK293T cells transfected with HA-HSF1 and Flag empty vector or Flag-Arc, were exposed to heat shock, and the cell lysates were immunoprecipitated using anti-Flag antibody. The immune-complex was then subjected to Western analysis with anti-HA antibody to detect HA-HSF1. As shown in Fig. 4a, Flag-Arc interacted with HSF1 only in cells treated with heat shock and not control cells, indicating that Arc/Arg3.1 binds to HSF1 in stressed cells. To exclude the non-specific binding by heat shock, we performed the same immunoprecipitation with Flag-Arc antibody in cells overexpressing HA-ezrin. As shown in Supplementary Fig. 5, Flag-Arc did not interact with HA-ezrin in either heat shock treated or untreated cells, confirming that Flag-Arc interacts with HA-HSF1 specifically. To elucidate whether the interaction between HSF1 and Arc/Arg3.1 is direct or indirect, we performed pull-down assay with GST and GST-tagged HSF1 and His-tagged Arc/Arg3.1 protein. As shown in Fig. 4b, His-Arc/Arg3.1 protein bound to GST-HSF1, not to GST control regardless heat shock treatment. This may be due to formation of GST dimers that would induce HSF1 oligomerization and allow for Arc/Arg3.1 interaction. This suggests that HSF1 interacts with Arc/Arg3.1 via direct binding.

We investigated how Arc/Arg3.1 affects the activation of HSF1. HEK293T cells overexpressing Flag empty vector or Flag-Arc were exposed to heat shock at 45°C for 15 min and recovered at 37°C for various time. HSF1 was immediately phosphorylated by heat shock as detected by the band up-shift, which returned to its original position after dephosphorylation after 7 h recovery. The kinetics of HSF1 phosphorylation by heat shock were identical between control and Flag-Arc overexpressed cells (Fig. 4c). This indicates that Arc/Arg3.1 does not affect the initial HSF1 phosphorylation by heat shock.

Activated HSF1 is known to form trimers and translocate from the cytosol to the nucleus. We investigated whether Arc/Arg3.1 inhibits the translocation of activated HSF1 into the nucleus. For this, we examined the subcellular localization of HSF1 in Flag empty vector and Flag-Arc transfected cells. HEK293T cells overexpressing Flag empty vector or Flag-Arc were exposed to heat shock and fractionated into cytosolic and nuclear fractions. As shown in Fig. 4d, HSF1 was present in the cytoplasm before heat shock, and translocated into the nucleus immediately after heat shock treatment. Arc/Arg3.1 overexpression did not affect the translocation of HSF1 into the nucleus. Intriguingly, overexpressed Arc/Arg3.1 is present in both cytosol and nucleus, and also translocated into the nucleus by heat shock treatment. Endogenous Arc/Arg3.1 induced by heat shock was localized in both of the cytosol and nucleus, as well (Fig. 4e). Prx6 and PARP were used as cytosolic marker and nuclear marker protein, respectively. To confirm that Arc/Arg3.1 did not affect the translocation of HSF1, we examined the localization of HSF1 and Flag-Arc in cells overexpressing Flag-Arc employing confocal microscopy. HEK293T and HeLa cells transfected with Flag empty vector and Flag-Arc were heat shock treated, and endogenous HSF1 and Flag-Arc were detected under confocal microscopy (Fig. 4f and Supplementary Fig. 6). Endogenous HSF1 was normally present in the nucleus, with some part dispersed in the cytosol. After heat shock treatment, HSF1 is mainly localized in the nucleus. Flag-Arc overexpression did not affect HSF1 localization in neither normal cells nor heat shock treated cells, but Flag-Arc was translocated to nucleus by heat shock and colocalized with HSF1. These findings indicate that Arc/Arg3.1 is also translocated to the nucleus along with activated HSF1 in response to heat shock, but did not affect the translocation of active HSF1 from the cytosol to the nucleus.

**Figure 5:**
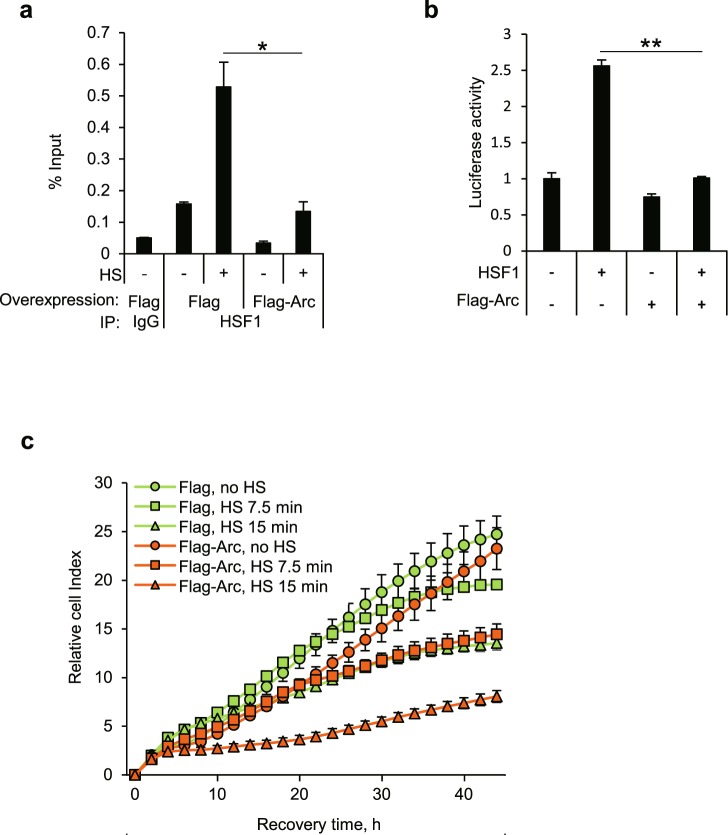
Arc/Arg3.1 reduces HSF1 affinity to HSE and interferes with cellular chaperone activity. (**a**) HEK293T cells were exposed to heat shock at 45°C for 15 min and crosslinked with formaldehyde. Chromatin immunoprecipitation using rabbit IgG or anti-HSF1 antibody was performed. Immunoprecipitated HSE region of *hsp70* was quantified using quantitative real-time PCR. (**b**) HEK293T cells were transfected with HA-HSF1 and *hsp*27-luciferase plasmed with empty vector or Flag-Arc vector. After 24 h, luciferase activity was measured. (**c**) HEK293T cells were transfected with Flag empty vector or Flag-Arc and heat shock treated at 45°C for the indicated times. Cells were plated in 96-well plate and cell growth was monitored under real-timed cell analyzer, xCELLigence. Data were presented as the means ± S.D. of triplicated experiments (t-test; *P < 0.05, **P < 0.01).

Since Arc/Arg3.1 did not affect the initial phosphorylation-dependent activation of HSF1 nor the translocation of HSF1 to the nucleus, we examined whether Arc/Arg3.1 affects the binding of activated HSF1 to HSE, employing chromatin immunoprecipitation (ChIP) assay using anti-HSF1 antibody. HEK293T cells overexpressing Flag empty vector or Flag-Arc, were exposed to heat shock at 45°C for 15 min and the cell lysates were crosslinked with formaldehyde and immunoprecipitated with rabbit IgG or anti-HSF1 antibody. The immunoprecipitated HSE of *hsp70* was quantified by quantitative real-time PCR. Heat shock treatment increased HSF1 binding to HSE in Flag empty vector transfected cells. However, the overexpression of Flag-Arc decreased HSF1-HSE binding in heat shock treated cells (Fig. 5a). We examined if HSF1 also binds to HSE of *Hsp27* gene in the same way but we could not detect any such binding (Supplementary Fig. 7a), but *Hsp27* mRNA was synthesized normally (Fig. 3a). Our inability to detect HSF1 binding to the HSE of Hsp27 gene might be because we may have missed the time point at which HSF1 bound to HSE of *Hsp27* gene. When we examined the induction kinetics of Hsp27 and Hsp70 mRNAs, we found that *Hsp27* mRNA was synthesized slower than *Hsp70* mRNA (Supplementary Fig. 7b,c). Another possibility is that HSF1 binding to the HSE of Hsp27 gene might be below the detection limit because the maximum *Hsp70* mRNA induction level was 47 fold, compared to 9 fold induction in *Hsp27* mRNA (Supplementary Fig. 7b,c). To confirm this, we performed Hsp27 reporter gene assay. We transfected HEK293T cells with HA-HSF1 and Flag empty vector or Flag-Arc with Hsp27-luciferase plasmid, and performed reporter gene assay. We found, as shown in Fig. 5b, that luciferase activity of Hsp27 was significantly decreased in cells overexpressing Flag-Arc, indicating that Arc/Arg3.1 inhibits the transcription of *Hsp27* again. These results indicate that Arc/Arg3.1 inhibits HSF1 binding to HSE by interacting with HSF1 under heat shock condition.

### Arc/Arg3.1 negatively regulates the viability of heat shock treated cells

Since Arc/Arg3.1 acts as a negative regulator of HSF1 resulting in decreased level of Hsps, we examined the effect of Arc/Arg3.1 overexpression on cellular chaperonic activity^28^. HEK293T cells transiently co-transfected with firefly luciferase and Flag empty vector, Flag-Arc or GFP-Hsp70. These cells were then exposed to heat shock at 45°C for 15 min to denature luciferase, and recovered at 37°C for the indicated times to renature and reactivate luciferase. At each time point, luciferase activities were measured. Luciferase activities were decreased by heat shock to less than 10% of luciferase activity of heat shock untreated and recovered during the recovery. As shown in Supplementary Fig. 8a,b, control cells transfected with Flag empty vector alone showed 65% reactivation of heat-denatured luciferase after 12 h recovery. Cells overexpressing GFP-Hsp70 enhanced the reactivation of luciferase activity over 80% in 12 h recovery as expected, whereas cells overexpressing Flag-Arc showed only ~45% recovery of luciferase activity. This indicates that overexpression of Arc/Arg3.1 inhibits cellular chaperonic activities.

Since Arc/Arg3.1 inhibits the production of Hsps mRNA and reduces cellular chaperonic activity, we investigated whether Arc/Arg3.1 affects cell survival after heat shock treatment. HEK293T cells transfected with Flag empty vector or Flag-Arc were exposed to heat shock at 45°C for 0, 7.5, 15 min and the cell survival monitored, employing a real-time cell analyzer, xCelligence, based on the conductivity of attached cells on the gold surface (Fig. 5c) and WST-1 cell proliferation assay (Supplementary Fig. 8c). Cells overexpressing Flag-Arc were more sensitive to heat shock and exhibited decreased cell survivals. These results demonstrate that Arc/Arg3.1 overexpression decreases cell survival in response to heat shock.

## Discussion

In the present study, we have shown that Arc/Arg3.1, transiently expressed in response to heat shock stresses just like Hsps, inhibits *hsp* gene activation by inhibiting the binding of HSF1 to HSE, and reduces cell survival in response to stress. Thus Arc/Arg3.1 acts as a novel negative regulator of activated HSF1 activity.

This study is the first to report on the novel induction and function of Arc/Arg3.1 in stress signaling pathway both in neuronal and non-neuronal cells. Heat shock, diamide, sodium arsenite and H_2_O_2_, are all HSR inducing stresses by inducing Hsps. They do it by inducing protein denaturation and misfolding in different ways. Diamide forms protein-protein disulfide bonds; sodium arsenite, used as a pesticide, is a blocker of cysteine residues; and H_2_O_2_, is an oxidant for cysteine and methionine residues. We found that the accumulation of abnormal proteins in cells with all the above stresses induces Arc/Arg3.1 as well as Hsps. Multiple studies have demonstrated that Arc/Arg3.1 can be naturally induced by glutamate in cortical neuron^29^, BDNF^30^ and PDGF^31^ in hippocampus, nociceptive simulation in spinal cord^32^, actin polymerization in amygdala^33^ and *Arc/Arg3.1* gene activated by a transcription factor, Egr1^31^. The relationship, if any, between neuronal- and heat shock mediated induction of Arc/Arg3.1 and the underlying molecular mechanism, should be further studied.

Arc/Arg3.1 is induced by various stresses including heat shock as just like Hsps, but is readily degraded during recovery in ubiquitin-proteasome system, unlike Hsps (Fig. 2). Half-life of Arc/Arg3.1 induced by heat shock is about 30 min, which is similar to that of induced by muscarinic cholinergic receptor stimulation (~37 min)^34^. The levels of Arc/Arg3.1 protein in neurons are tightly regulated and it is removed by proteasome-mediated degradation by Triad3A E3-ligase^35^. A recent report demonstrates that GSK3α/β-catalyzed Arc/Arg3.1 phosphorylation and ubiquitination regulate the duration of Arc/Arg3.1 expression and function in dendritic spine^36^. Since Arc/Arg3.1 is induced and degraded faster in response to heat shock in thermotolerant cells than in normal cells, just like canonical Hsp expression, further studies are required to understand the regulation of Arc/Arg3.1 expression in neuronal and non-neuronal systems. Despite the similarity in their induction, their roles are different in that Hsps serve as chaperones in protein synthesis, in refolding of denatured proteins, and serving as co-factors of UPS, while the role of Arc/Arg3.1 seems to reduce HSR through inhibition of HSF1. Possibly, Arc/Arg3.1 is another regulation system for HSF1 in HSR in addition to the chaperones.

Activation of HSF1 is a major feature of HSR. Since HSR is a cellular response to stresses, one would expect that there would be mechanism to turn it off when the stresses are removed. Attenuation of HSF1 binding to HSE is regulated by post-translational modifications of HSF1 such acetylation of Lys80, phosphorylation (Ser303) dependent sumoylation of Lys298 and phosphorylations of Ser residues^3^. In addition, binding of Hsp70 and Hsp40 reduces transactivation capacity of HSF1^37^. In this study, Arc/Arg3.1 bound to HSF1 as Hsp70 and Hsp40 do, inhibited its binding to HSE resulting in inhibition of HSF1 transcription. Arc/Arg3.1 function on HSF1 is distinguished from those of Hsp70 and Hsp40.

HSF1 was initially believed to be a transcription factor that upregulates genes encoding heat shock proteins (HSPs), also called molecular chaperones, which assist in refolding or degrading injured intracellular proteins. However, recent accumulating evidence indicates multiple additional functions for HSF1 beyond the activation of HSPs, as non-HSP-related target genes have been identified. Through controlling these targets, HSF1 acts in diverse stress-induced cellular processes via distinct molecular mechanisms, including the endoplasmic reticulum unfolded protein response and ubiquitin–proteasome system, multidrug resistance, autophagy, apoptosis, immune response, cell growth arrest, differentiation underlying developmental diapause, chromatin remodelling, cancer development, and ageing. Hence, HSF1 emerges as a major orchestrator of cellular stress response pathways^38,39^, and is suggested as a cancer biomarker and therapeutics^40^. Accumulation of molecular chaperones induced via HSF1 attributes to increases in folding demand in cancer, or to the evolution of new mechanisms for induction of HSR in quickly adapting cancer cells^41^. Since we found that Arc/Arg3.1 overexpression inhibits HSF1 transcriptional activities, we can suggest that Arc/Arg3.1 can be employed as a negative regulator of HSF1 in cancer treatment. Further studies for identifying the downstream targets of Arc/Arg3.1 should be done to explore the underlying mechanism in cancer cells.

In summary, this study identifies Arc/Arg3.1 as a novel Hsp because of the similarity of its induction to other Hsps, but one which is rapidly degraded in ubiquitin proteasome system. The half-life of Arc/Arg3.1 is shorter than 30 min, the shortest protein half-life compared to those of most proteins^28^. Arc/Arg3.1 functions in negative feedback loop of HSR, impeding activated HSF1 functions. Whether the short half-life of Arc/Arg3.1 is necessary for its function in the feedback loop can only be answered by further studies.

## Materials and Methods

### Cell lines

Radiation induced mouse fibrosarcoma cell line, RIF-1 and its thermotolerant variant, TR-RIF-1 (TR) cell lines (gifts from Dr. Hahn G. M.) were grown in RPMI1640, supplemented with 10% fetal bovine serum (FBS), 100 μg/mL streptomycin, 100 units/mL penicillin G, 3.75 μg/mL sodium bicarbonate, and 0.11 μg/mL sodium pyruvate at 37°C in a 5% CO_2_-containing humidified incubator. HeLa, HEK293T and SH-SY5Y cells were purchased from ATCC and maintained following the manufacturer’s protocol. Wild type mouse embryonic fibroblast, HSF1(+/+) MEF, and HSF1 knockout MEF, HSF1(−/−), cell lines (gifts from Ivor J. Benjamin) were grown in high glucose Dulbecco’s Modified Eagle Medium (DMEM), supplemented under same condition.

### Antibodies and reagents

Monoclonal Arc antibody (E-7 & C-7), monoclonal HSP27 antibody, monoclonal α tubulin antibody, monoclonal β-actin antibody and monoclonal GST antibody were purchased from Santa Cruz: polyclonal GAPDH antibody, monoclonal HA antibody and polyclonal Prx6 antibody from Ab Frontier, Inc.: monoclonal Hsc/Hsp70 antibody, monoclonal HSF1 antibody and monoclonal PARP-1 antibody from Enzo: monoclonal FLAG antibody from Sigma Aldrich: monoclonal Lamin B antibody from Calbiochem. Hydrogen peroxide was purchased from Samchun Pure Chemical Co. and diamide, sodium arsenite and cycloheximide from Sigma Aldrich.

### Plasmids

Human Arc/Arg3.1 was subcloned into pFlag-CMV-2 vector. For cloning the Flag-tagged human Arc DNA, human Arc cDNA was prepared by PCR using the sense primer 5′-GGAATTCGAGCTGGACCACCGG-3′ and antisense primer 5′-CGGGATCCCTACTCGGGCTGGGT-3′. The PCR products and vectors were digested using EcoRI/BamHI and were ligated using pGEM T-Easy vector system (Promega). pCytluc (pRSVLL/V) encoding cytoplasmic luciferase was a gift from Dr. S. Subramani (University of California, San Diego, USA). GFP-tagged Hsp70 plasmid was a gift from Dr. Yun Sil Lee (Ewha Womans University, Korea).

### Transfection

HeLa and HEK293T cells were transiently transfected with expression plasmids using TransIT-LT1 (Mirus). HEK293T cells were transfected with expression plasmids using the effectene transfection reagent (Qiagen).

### Quantification of hsp27, hsp70 and hArc mRNA using RT-qPCR

Total RNA was isolated using an RNeasy minikit (Qiagen) and cDNA was prepared using SuperScript II RT (Invitrogen) according to the manufacturer’s protocol. Synthesized cDNA was subjected to real-time PCR analysis using SYBR green qPCR master mix (Applied Biosystems) and AB7300 real-time qPCR machine (Applied Biosystems). The following primers of human genes were used: hsp27 gene: forward primer, 5′-CATCCCAGTCACCTTCGAGT-3′; reverse primer, 5′-CTTTACTTGGCGGCAGTCTC-3′, hsp70 gene: forward primer, 5′-CCGAGAAGGACGAGTTTGAG-3′; reverse primer, 5′-CTGGTACAGTCCGCTGATGA-3′, hArc gene: forward primer, 5′-GGAGTACTGGCTGTCCCAGA-3′; reverse primer, 5′-ACTCCACCCAGTTCTTCACG-3′ and GAPDH gene: forward primer, 5′-AAG GTC ATC CCT GAG CTG AA-3′; reverse primer, 5′-TGC TGT AGC CAA ATT CGT TG-3′. Standard curve method was used for quantification.

### siRNA

Negative control siRNA (AccuTargetTM SN-1001-CFG) and human Arc/Arg3.1 siRNAs (predesigned siRNA #1007182 and 1007189) were purchased from Bioneer (Korea).

### ChIP assay

ChIP assay was performed using slightly modified EZChIP kit (Merck Millipore). Immunoprecipitation in HEK293 cells was carried out using polyclonal anti-HSF1 (Enzo). Immunoprecipitated DNA was analyzed by quantitative real-time PCR analysis using primers for heat shock element region of *hsp70* gene: forward, 5′–CACTCCCCCTTCCTCTCAG–3′; reverse, 5′–TTCCCTTCTGAGCCAATCAC–3′.

### Nuclear/cytosolic fractionation

Cells were lysed in hypotonic buffer (10 mM HEPES, pH 7.9, 1.5 mM MgCl_2_, 10 mM KCl, protease inhibitor cocktail (Sigma)) by passing 31 G syringe 10 times and incubation on ice for 30 min. After centrifugation at 4,000 rpm for 25 min, supernatant (cytosolic fraction) was removed. The pellet (nuclear fraction) was washed with hypotonic buffer twice.

### Confocal microscopy

HEK293T cells were plated on the glass coverslip 24 h before transfection. Cells were transfected with 0.25 μg of Flag or Flag-Arc. After 24 h, cells were treated with heat shock at 45°C for 15 min and immediately fixed with 4% paraformaldehyde. Cells were permeabilized with 0.1% Triton X-100, blocked with blocking solution (3% bovine serum albumin; 0.2% Tween 20 and 0.2% gelatin) and incubated with anti-Flag (20 µg/mL) and anti-HSF1 (1:100 dilution) primary antibodies for 2 h at 37°C. Alexa Fluor 488 conjugated goat anti-mouse (1:100) and Alexa Fluor 568 conjugated goat anti-rabbit (1:100) secondary antibodies (Invitrogen) were used to visualize Flag-Arc and HSF1, respectively. Coverslips were mounted using antifading solution containing DAPI (Molecular Probes) and images were obtained using LSM 880 Airy scan confocal microscope (Zeiss).

### Immunoprecipitation

Cells were lysed in IP buffer (50 mM Tris-Cl, pH 7.4, 150 mM NaCl, 1 mM EDTA, 1% triton X-100) and hypotonic buffer (10 mM HEPES, 1.5 mM MgCl_2_, 10 mM KCl, 1% triton X-100) 3:1 mixture supplemented with protease inhibitor cocktail (Sigma). Cells were passed 31 G syringe 10 times and centrifuged at 12,000 rpm for 5 min. The supernatant was incubated with anti-Flag antibody for 2 h at 4°C while gently rotating. The lysate-antibody complexes were incubated with protein-G sepharose 4 Fast Flow beads for another 1 h at 4°C. Thereafter, the precipitated beads were washed five times with washing buffer (50 mM Tris-Cl, pH 7.4, 150 mM NaCl, 1 mM EDTA, and 0.5% Nonidet P-40) and three times more with washing buffer without detergent. Gel sample buffer was added to the beads and boiled at 95°C for 10 min to elute the immune-complexes. The samples were analyzed by Western analysis.

### *In vitro* protein binding assay

Cytosol of GST-hHSF1 expressed *E. coli* was incubated with glutathione-agarose beads in PBS for 3 h at 4°C. The beads were washed three times with PBS containing 0.1% Triton X-100. GST-hHSF1 bound to glutathione-Agarose beads and his-tagged hArc/Arg3.1 in binding buffer (20 mM MOPS, pH 7.2, 100 mM KCl, 0.5% Tween and 2 μg/mL aprotinin) were incubated for 3 h at 4°C with rocking. The beads were washed five times with binding buffer and analyzed by Western analysis using polyclonal anti-GST and anti-Arc antibody.

### Luciferase reactivation assay

Cells were transiently transfected with pCytluc together with GFP-tagged Hsp70 or Flag-tagged Arc/Arg3.1. Twenty-four hours after transfection, the cells were transferred into tissue culture dishes in medium with 20 μg/mL cycloheximide for 30 min at 37°C to stop expression of luciferase. Luciferase was inactivated by heating the cells at 45°C for 15 min and recovered at 37°C for various times. Luciferase activities of the harvested cells were measured using luciferase assay kit (Promega).

### Cell proliferation assay

HeLa cells were exposed to heat shock at 45°C for 0, 7.5 and 15 min and recovered at 37°C for 3 h in fresh media. For real-time cell analyzer analysis, 10,000 cells were plated in 96 well E-plate with 200 μL of media and cell growth was monitored by measuring electrical impedance every 15 min under xCELLigence RTCA SP system (Roche Applied Science). WST-1 cell proliferation assay was performed using WST-1 (Roche). HEK293T cells were transfected with Flag or Flag-hArc vector and treated with heat shock at 45°C for 0, 7.5 and 15 min, then cells were dispensed in 96-well microtiter plates and incubated at 37°C with fresh media for 1 day, and cell survival was measured using WST-1 solution. The cleavage of WST-1 to formazan by metabolically active cells was quantified by scanning the plates in a microtiter plate reader at 440 and 620 nm (reference wavelength). The test medium was used as the background control. Three independent sets of experiments that were performed in triplicate were evaluated. The viability of the treated cells was normalized to the untreated control cells.

## Supplementary information

Supplementary Information
