# Supplementary material for: Activity-Regulated Cytoskeleton-Associated Protein (Arc/Arg3.1) is Transiently Expressed after Heat Shock Stress and Suppresses Heat Shock Factor 1
Source: Sci Rep. 2019 Feb 22;9:2592. doi: 10.1038/s41598-019-39292-1 (PMC6385231; doi:10.1038/s41598-019-39292-1)
Supplement: Supplementary file 1 — Supplementary Information [file 41598_2019_39292_MOESM1_ESM.pdf]

Supporting Information

Activity-Regulated Cytoskeleton-Associated Protein (Arc/Arg3.1) is Transiently Expressed after Heat Shock Stress and Suppresses Heat Shock Factor 1

A Young Park[1*], Yeon Seung Park[1*], Dami So[1*], In-Kang Song[1], Jung-Eun Choi[1],

Hee-Jung Kim[1,2†], Kong-Joo Lee[1†]

*These authors contributed equally to this work.

[1]College of Pharmacy and Graduate School of Pharmaceutical Sciences, Ewha Womans University, Seoul, Korea 03760

[2]Current address: Spark biopharma, #203-207A, Seoul National University, 1 Gwanak-ro, Gwanak-gu, Seoul, Korea 08826

[†]Corresponding authors: Kong-Joo Lee, Tel: 82-2-3277-3038, Fax: 82-2-3277-3760, e-mail address: kjl@ewha.ac.kr; Hee Jung Kim, hjkim@sparkbio.co.kr

S Figure 1

a

Definition	Transcript	Symbol	Heat shock								MG132							
			RIF-1				TR				RIF-1				TR			
			C	4 h	12 h	24 h	C	4 h	12 h	24 h	C	2.5 h	8 h	18 h	C	2.5 h	8h	18h
Arc/ARG3.1	NM_018790.1	Arc	1.00	**37.9**	**88.8**	**12.6**	1.00	**38.0**	1.57	-1.34	1.00	**2.80**	-1.12	-1.38	1.00	1.59	-1.19	1.07
early growth response 1 (Egr1)	NM_007913.2	Egr1	1.00	**5.00**	**7.27**	**2.80**	1.00	**3.64**	-1.22	-1.27	1.00	**-4.67**	**-2.51**	-1.77	1.00	**-6.41**	**-3.02**	-1.72
FBJ osteosarcoma oncogene (Fos)	NM_010234.2	Fos	1.00	**2.70**	**7.89**	1.96	1.00	**5.42**	-1.52	-1.37	1.00	1.48	-1.14	-1.08	1.00	1.33	-1.21	-1.03
FBJ osteosarcoma oncogene B (Fosb)	NM_008036	Fosb	1.00	1.38	**5.10**	1.48	1.00	**4.72**	-1.01	-1.10	1.00	-1.13	1.02	1.11	1.00	-1.39	-1.15	-1.42
Jun oncogene (Jun)	NM_010591.1	Jun	1.00	1.72	**2.06**	-1.60	1.00	**2.61**	1.09	-1.22	1.00	-1.43	-1.81	**-2.28**	1.00	-1.69	-1.49	**-2.13**
Jun-B oncogene (Junb)	NM_008416.1	Junb	1.00	-1.25	**3.70**	1.15	1.00	**7.01**	1.30	**2.07**	1.00	1.38	-1.04	1.11	1.00	1.70	-1.35	-1.12

b

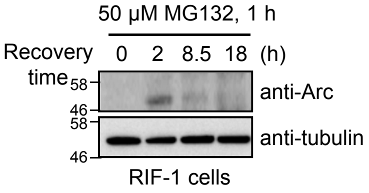

Supplementary Figure 1. (a) Gene list of immediate early genes (IEGs) induced by heat shock at 45°C for 30 min and recovered at 37°C for indicated times and by 50 μM MG132 treatment for 1 h in RIF-1 and TR cells compared to control cells. The results were extracted from microarray analysis obtained from previous results (Kim, H. J. et al. *PLoS One.* 6, e20252 (2011)). (b) RIF-1 cells were treated with 50 μM MG132 for 1 h as done in Figure 2a-c. Cells were recovered for the indicated times with fresh media. Arc/Arg3.1 was analyzed by Western analysis using anti-Arc/Arg3.1 antibody. As loading controls, tubulin levels were detected using anti-tubulin antibody.

S Figure 2

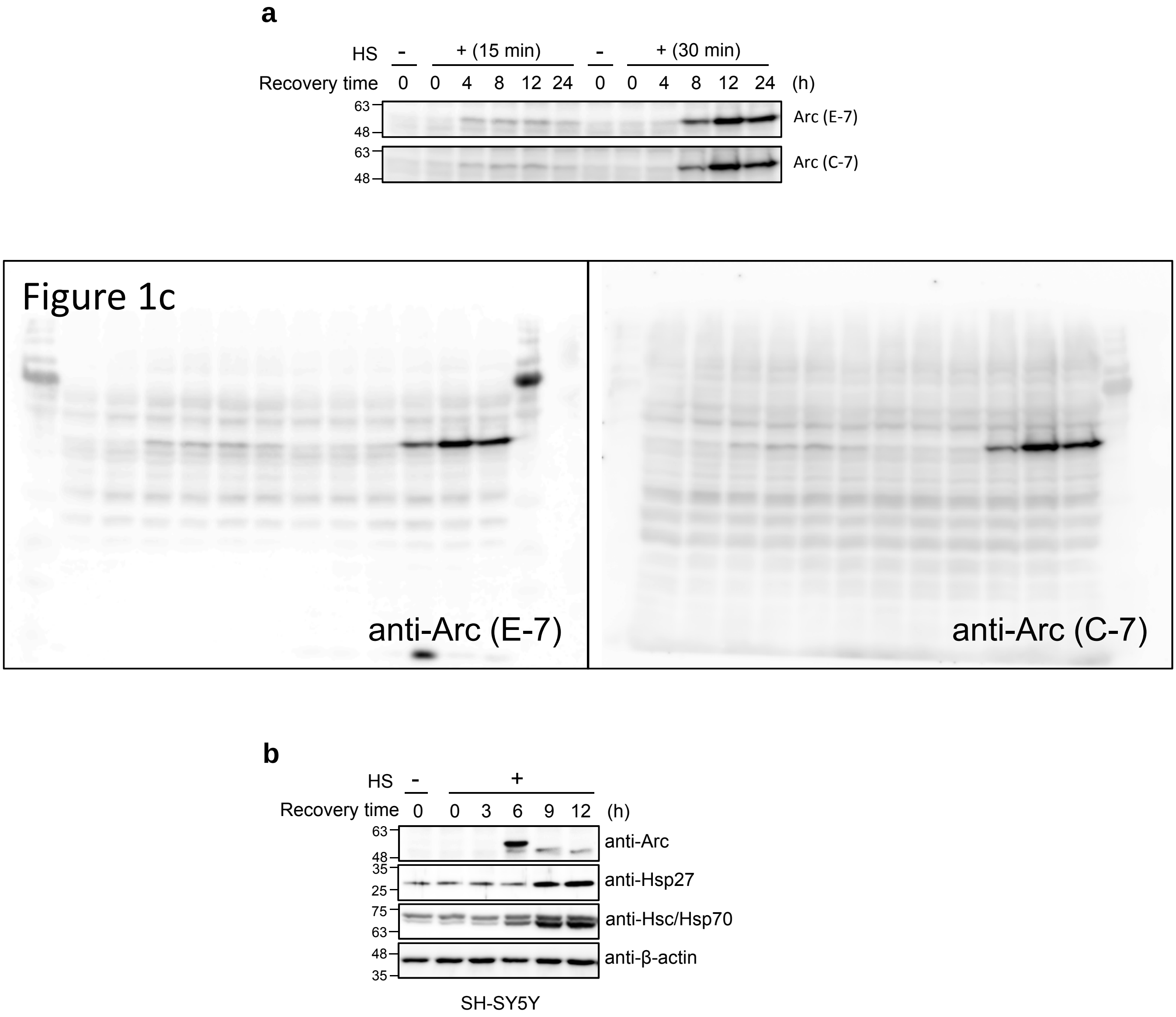

Supplementary Figure 2. (a) **Detection of Arc/Arg3.1 with different antibodies against Arc/Arg3.1.** HEK293T cells were exposed to heat shock at 45°C for 15 and 30 min, and recovered at 37°C for various times as shown in Figure 1c. Arc/Arg3.1 in same samples were detected with Western analysis using different antibodies (Santa Cruz, anti-Arc, E-7 and C-7). Bottom panel is the whole image of Western blot. No discernible differences between these two antibodies were detected. (b) Neuroblastoma SH-SY5Y cells were exposed to heat shock at 45℃ for 20 min and recovered at 37℃ for the indicated times. Arc/Arg3.1, Hsc/Hsp70 and Hsp27 were detected by Western blot analysis with their specific antibodies. As loading controls, β actin levels were detected using anti-β actin antibody.

S Figure 3

a

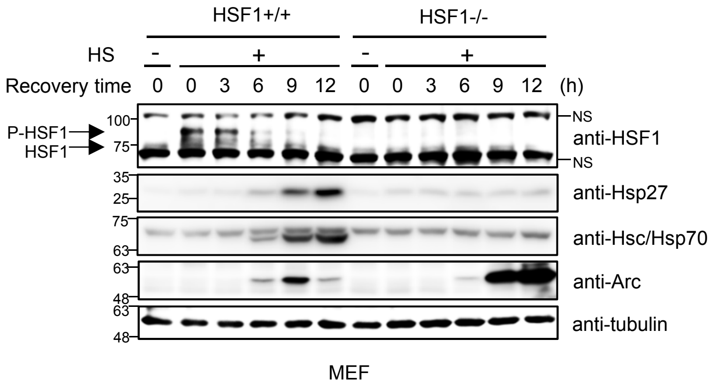

b

ARC_1 promoter sequence (chr8:142614467-142614526)

: ccacgggcctcgctggctgcataaagagccggcggccaggactcagcgcagagctcgggc

HSE consensus sequence:

nTTCnnGAAnnTTCn

Supplementary Figure 3. (a) HSF1 does not affect the expression of Arc/Arg3.1 in response to heat shock. MEF WT cells and MEF cells knocking down HSF1 were exposed to heat shock at 45°C for 20 min and recovered at 37°C for the indicated times. HSF1, Arc/Arg3.1, Hsp27 and Hsc/Hsp70 were detected by Western blot analysis with their specific antibodies. As a loading control, tubulin level was detected using anti-tubulin antibody. NS; non-specific band. **(b) There are no similar sequence of HSEs (heat shock elements) in the ARC promoter region.** Bioinformatics analysis was performed by comparing the sequences of Arc promoter region with heat shock element (HSE), which were provided by GeneCards and EPDnew (the Eukaryotic Promoter Database).

S Figure 4

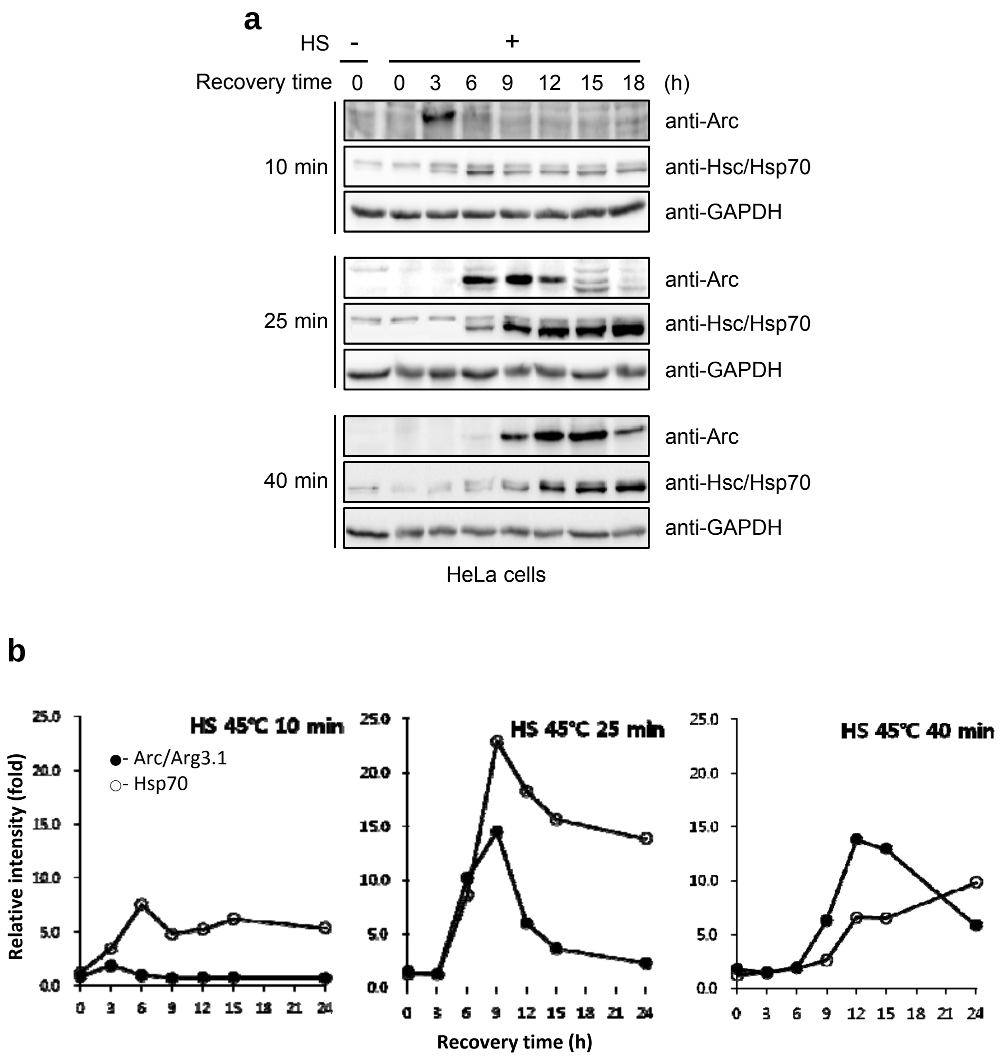

Supplementary Figure 4. HeLa cells were exposed to heat shock at 45°C for 10, 25, and 40 min and recovered for the indicated times in fresh media. Arc/Arg3.1 and Hsc/Hsp70 were detected by Western blot analysis with their specific antibodies. As a loading control, GAPDH level was detected using anti-GAPDH antibody. Arc/Arg3.1 and Hsc70/Hsp70 bands were quantified and normalized to the amount of GAPDH and showed graphs.

S Figure 5

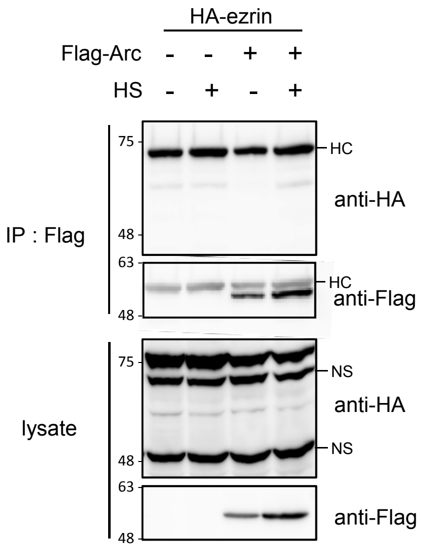

Supplementary Figure 5. HA-ezrin did not bind to Flag-Arc. HEK293T cells were transfected with HA-ezrin and Flag empty vector or Flag-Arc. After exposure to heat shock at 45°C for 15 min, immunoprecipitation was performed using anti-Flag antibody as done in Figure 4A. Protein complex was analyzed by Western analysis using anti-HA and anti-Flag antibodies. HC; antibody heavy chain, NS; non-specific band.

S Figure 6

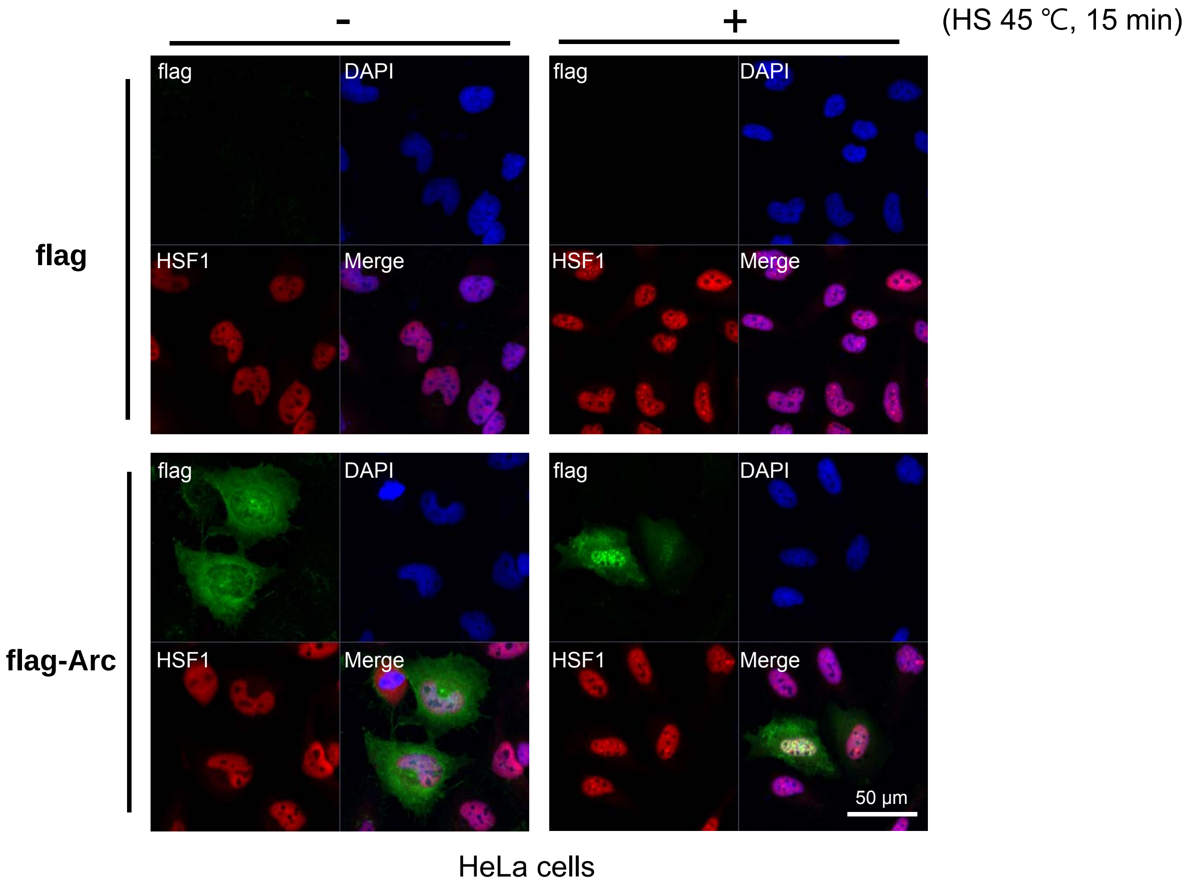

Supplementary Figure 6. HeLa cells were plated on the glass coverslip 24 h before transfection. Cells were then transfected with Flag or Flag-Arc. After 24 h, cells were treated with heat shock at 45℃ for 15 min and visualized Flag-Arc (green), HSF1 (red) and nucleus (blue) under confocal microscopy. All of the Western blot results were selected representative data from more than duplicated results.

S Figure 7

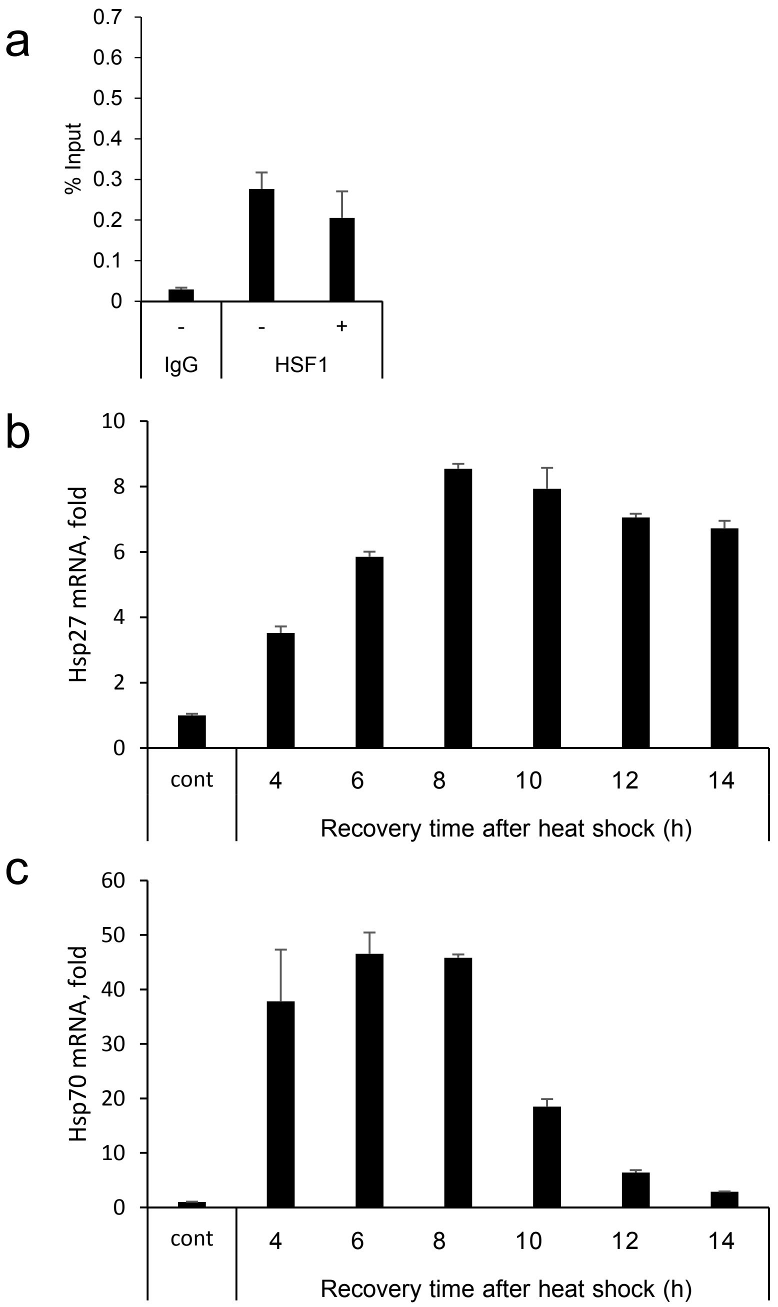

Supplementary Figure 7. HSF1 did not bind to the HSE of hsp27 immediately after heat shock. (a) Cells were exposed to heat shock at 45°C for 15 min and crosslinked with formaldehyde. Chromatin immunoprecipitation using rabbit IgG or anti-HSF1 antibody was performed. Immunoprecipitated HSE region was quantified using quantitative real-time PCR using primers forward, 5′–CAACCTGTCTGGCTCTGTCC–3′; reverse, 5′–GGCAATGACCCGTTTGAGG–3′. (b, c) HEK293T cells were heat shock treated at 45°C for 15 min and recovered for the indicated times at 37 °C. *hsp27* mRNA (b) and *Hsp70* mRNA (c) were analyzed using RT-qPCR.

S Figure 8

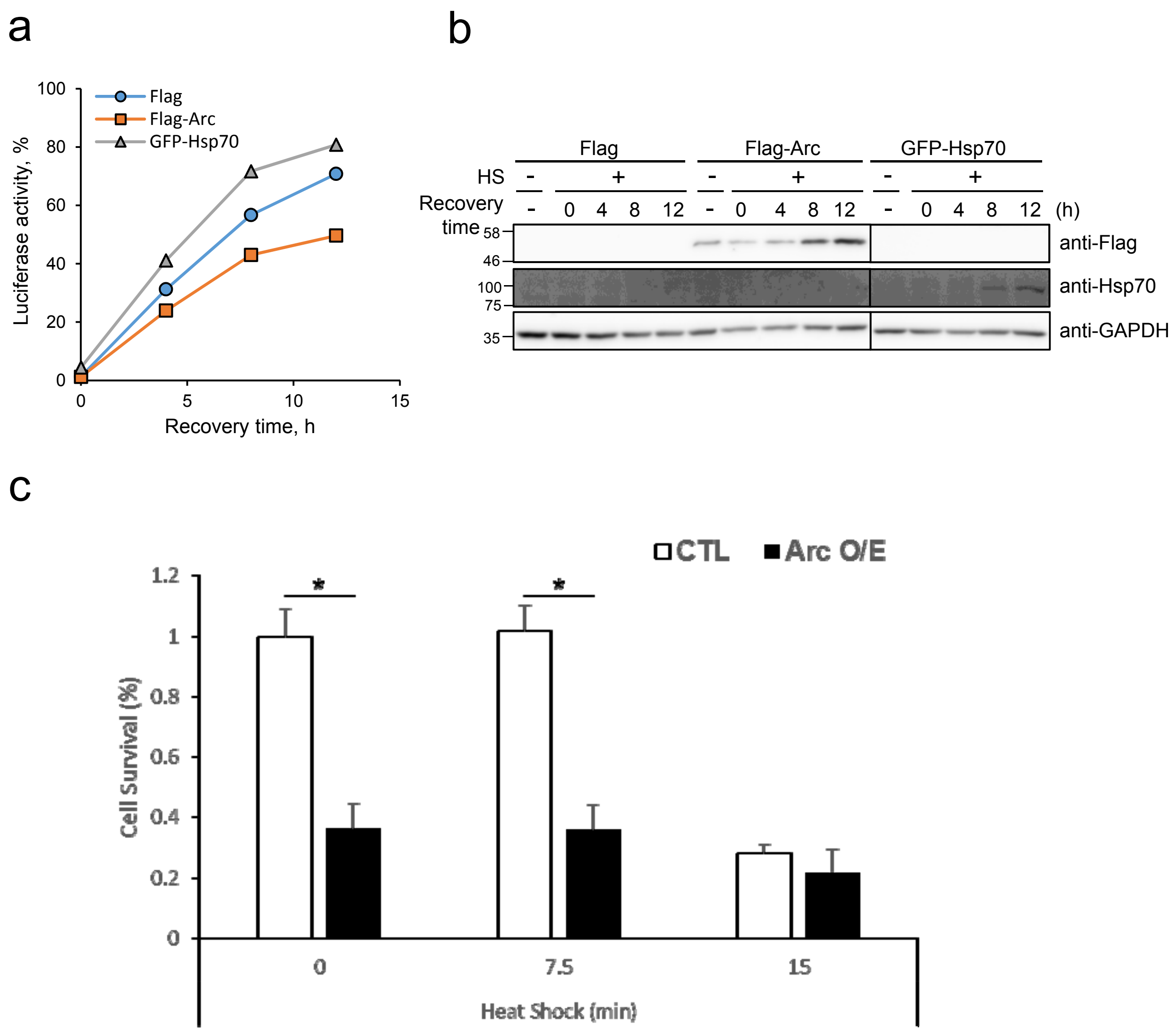

Supplementary Figure 8. (a) Arc/Arg3.1 interferes cellular chaperone activity. HEK293T cells were transfected with pCytluc (encoding cytosolic luciferase) together with Flag, Flag-Arc or GFP-Hsp70 vectors. After pretreatment with cycloheximide (20 μg/mL) for 30 min, luciferase was inactivated by heating the cells at 45°C for 15 min. During a subsequent recovery period at 37°C, luciferase activity at each time point was measured. (b) Overexpression of Flag-Arc and GFP-Hsp70 was confirmed by Western analysis. (c) HEK293T cells transfected with Flag empty vector or Flag-hArc vector were exposed to heat shock at 45℃ for indicated times. Cell were plated in 96-well plate, incubated at 37℃ for 1 day, and cell survival was measured using WST-1 proliferation assay. Data were presented as the means ± S.D. of triplicated experiments (t-test; *P<0.05).

S Figure 9

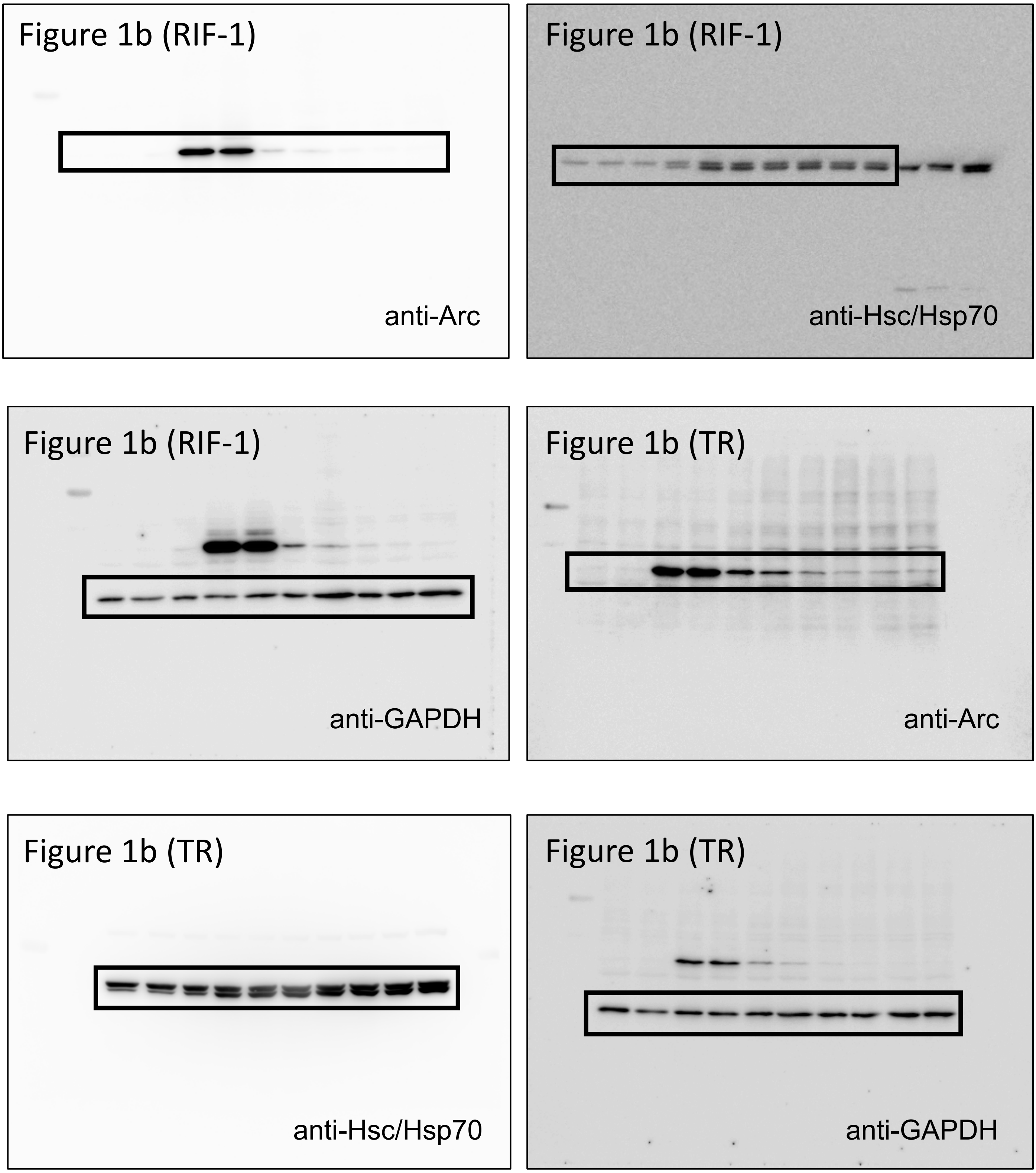

Supplementary Figure 8. Full size gels used in Figure 1-4.

S Figure 9

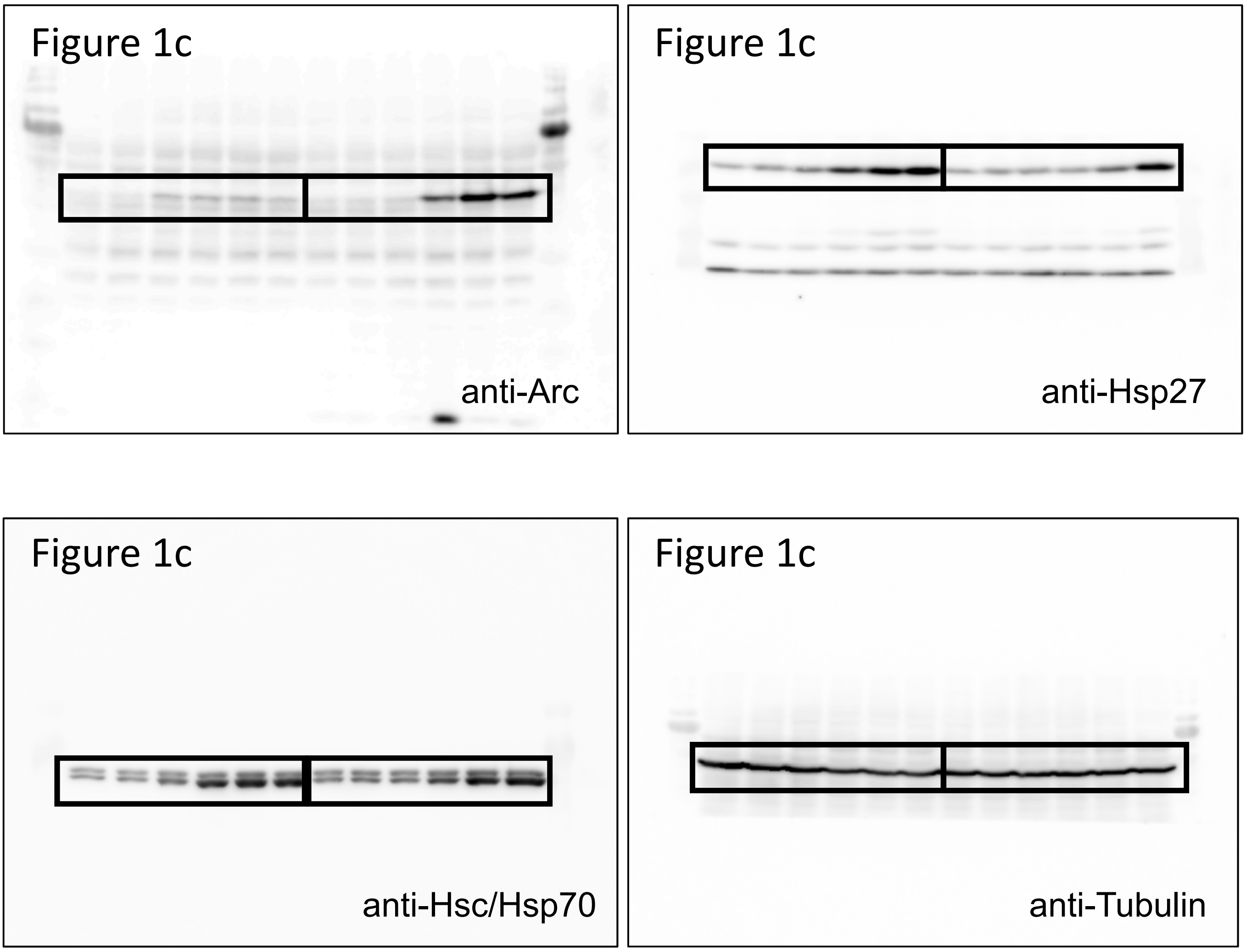

Supplementary Figure 8. Full size gels used in Figure 1-4.

S Figure 9

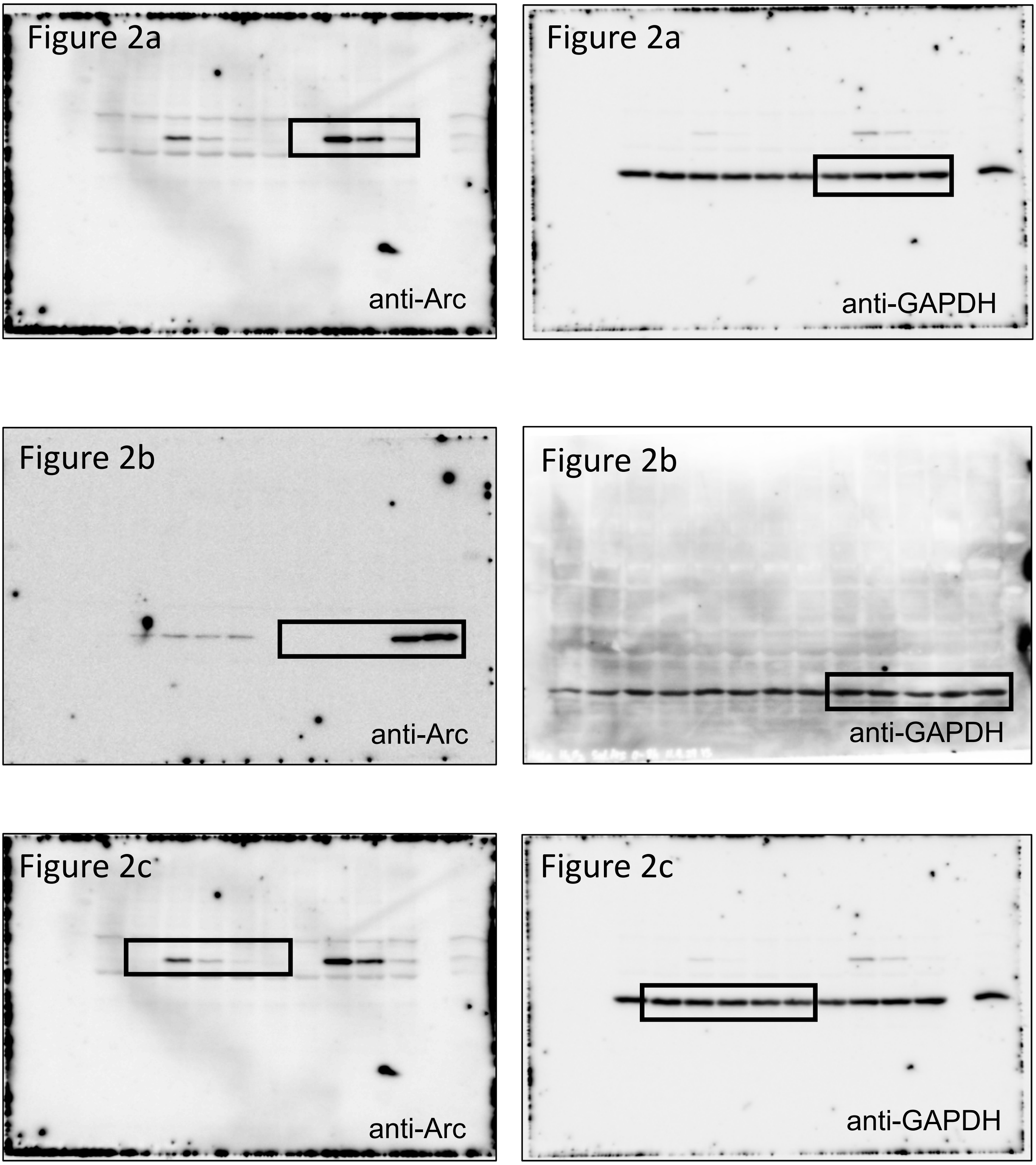

Supplementary Figure 8. Full size gels used in Figure 1-4.

S Figure 9

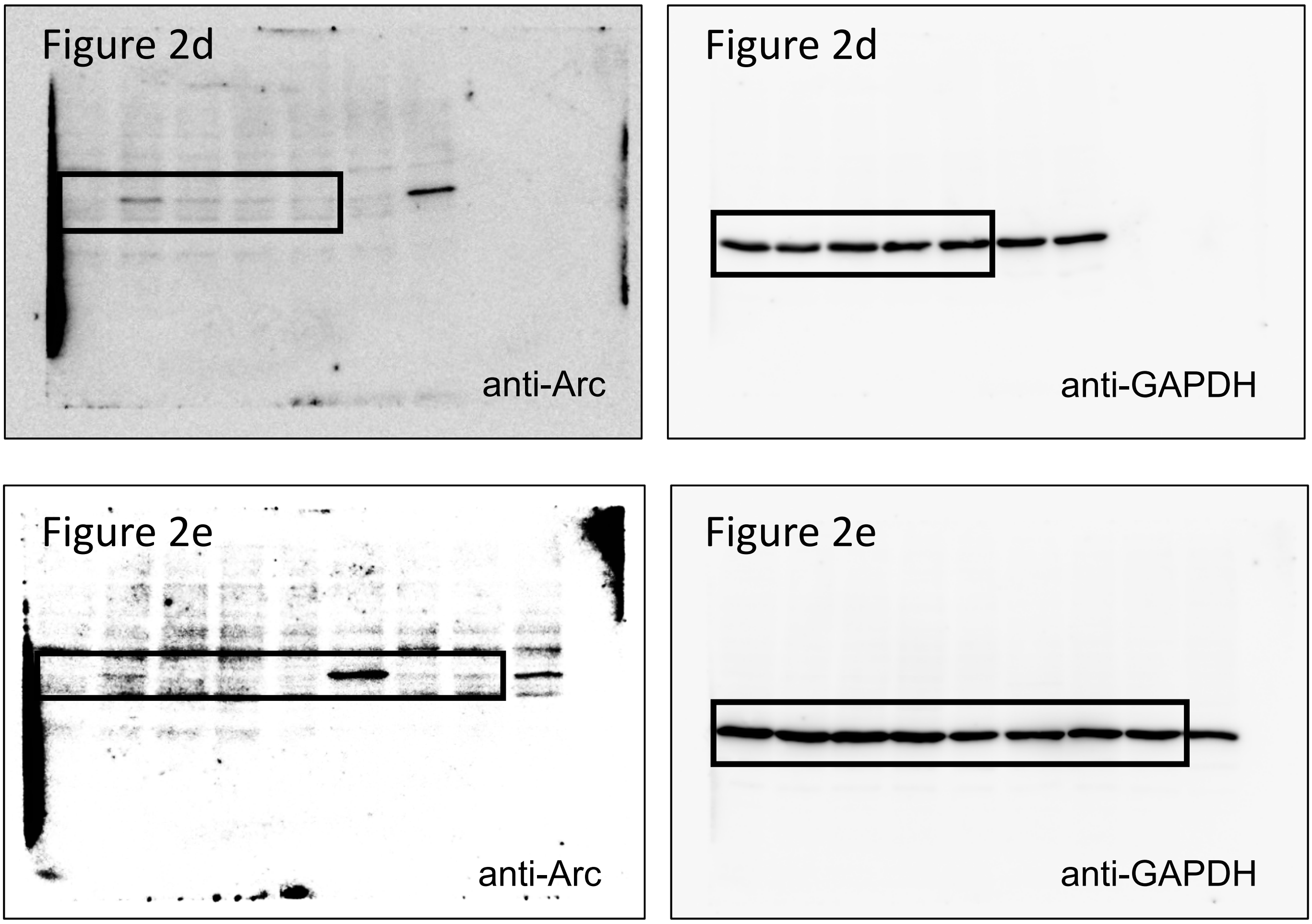

Supplementary Figure 8. Full size gels used in Figure 1-4.

S Figure 9

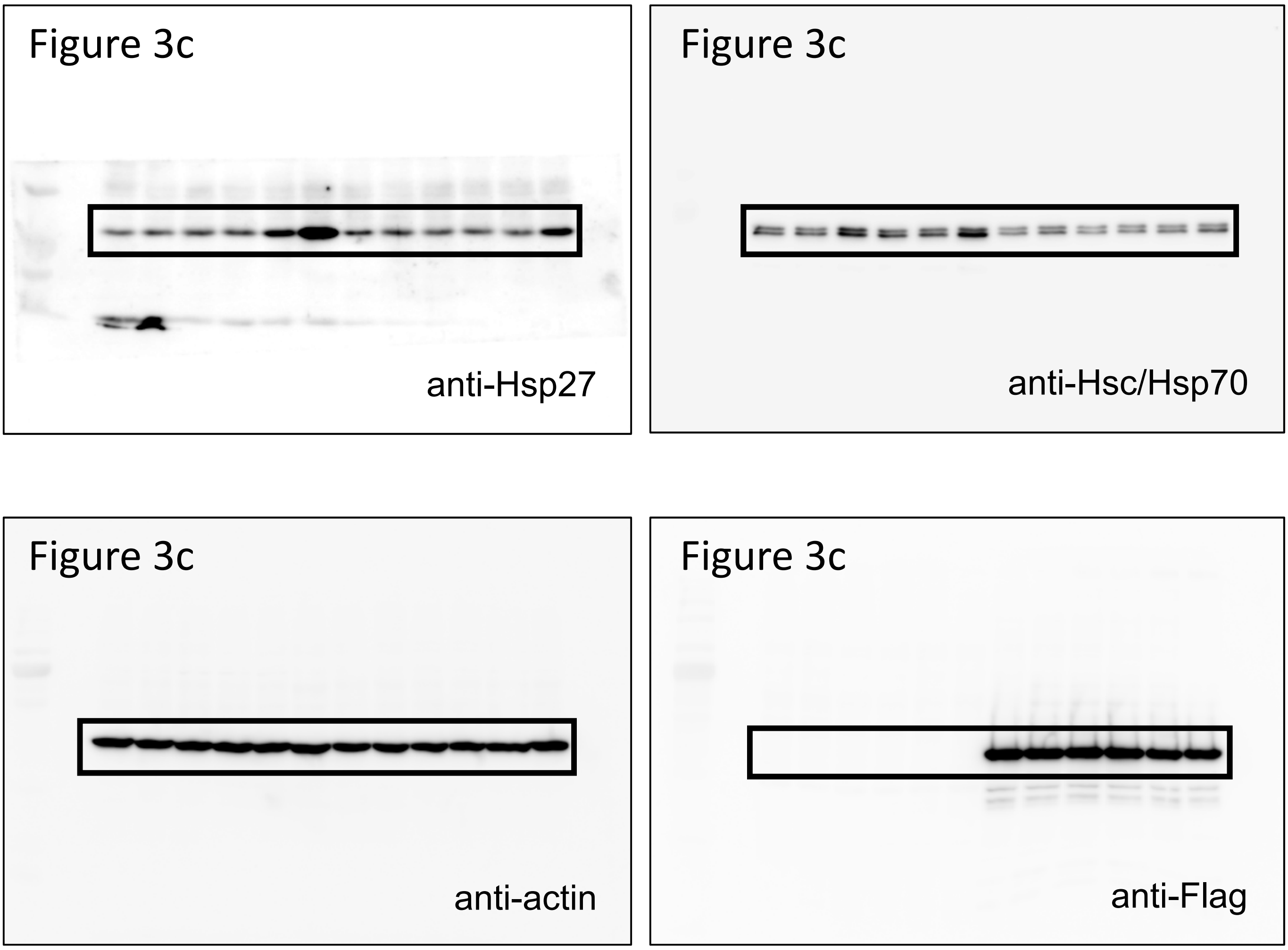

Supplementary Figure 8. Full size gels used in Figure 1-4.

S Figure 9

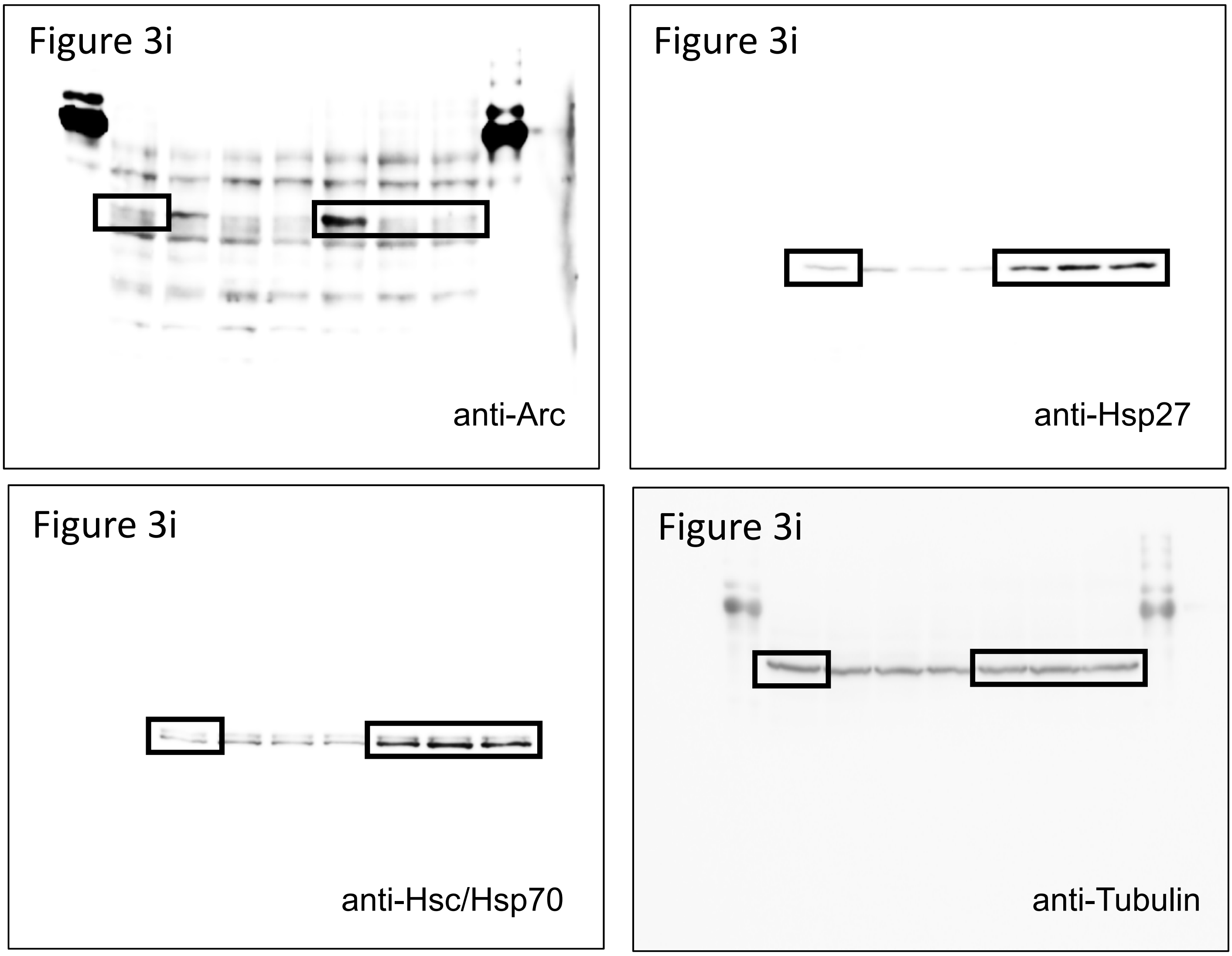

Supplementary Figure 8. Full size gels used in Figure 1-4.

S Figure 9

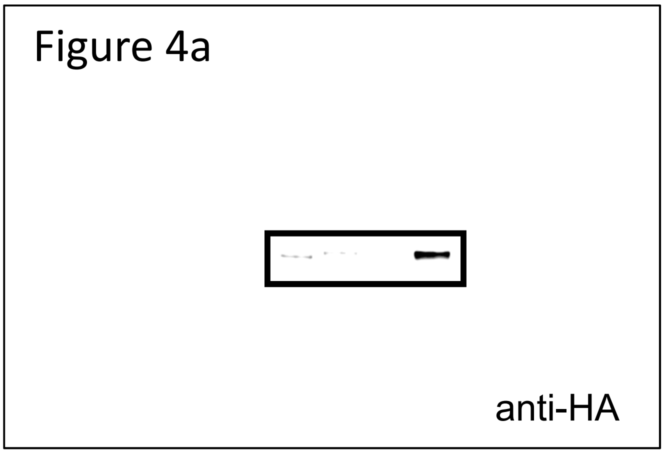

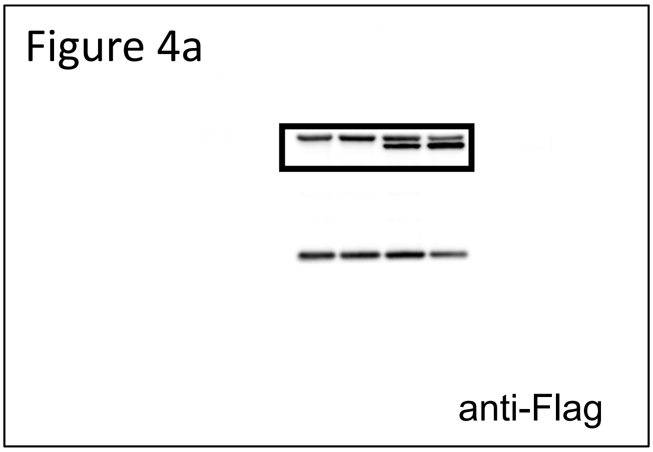

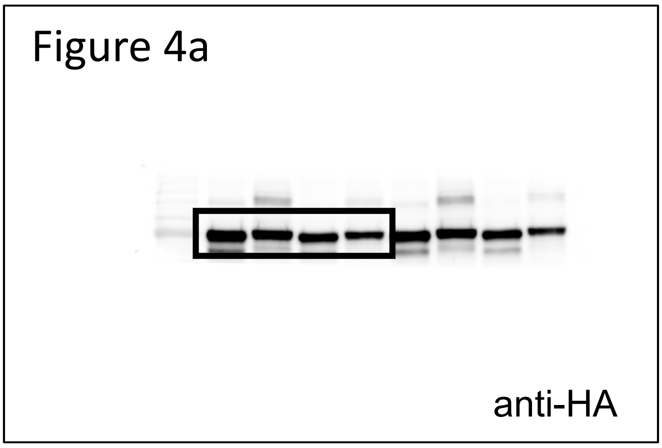

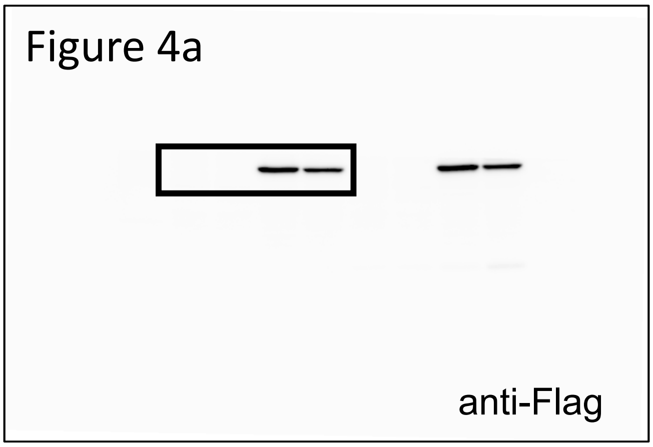

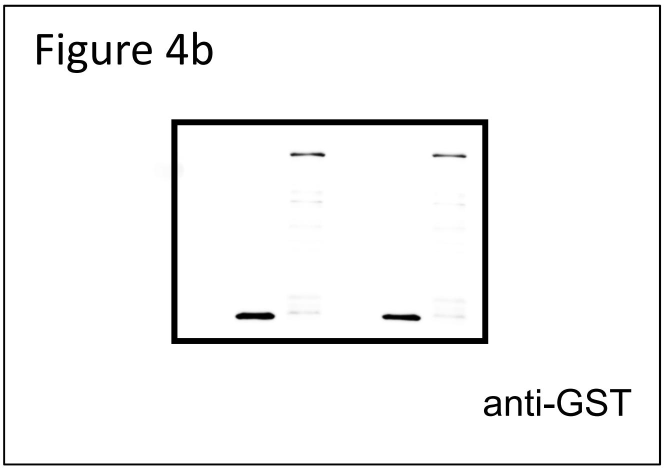

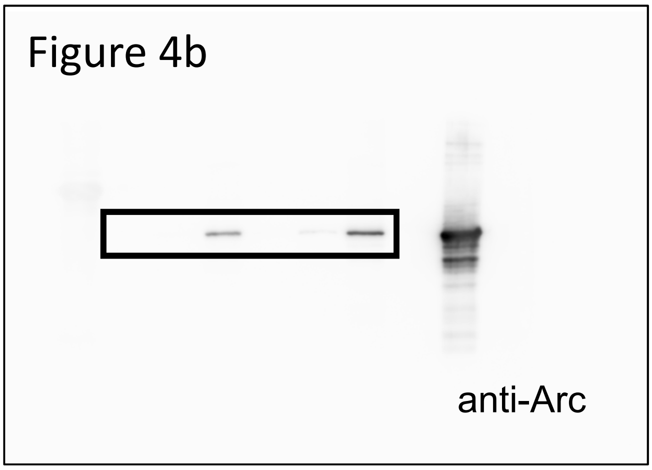

Supplementary Figure 8. Full size gels used in Figure 1-4.

S Figure 9

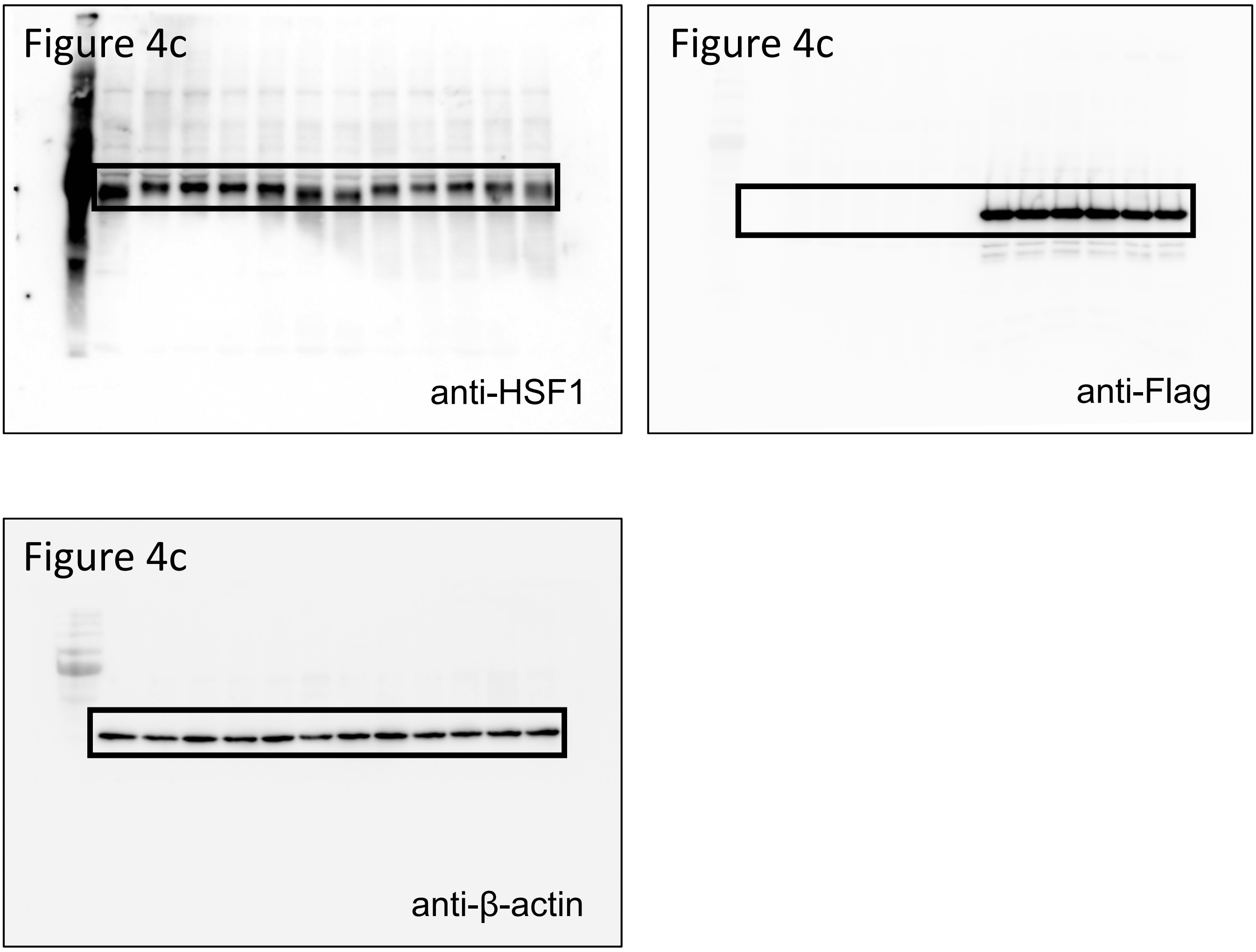

Supplementary Figure 8. Full size gels used in Figure 1-4.

S Figure 9

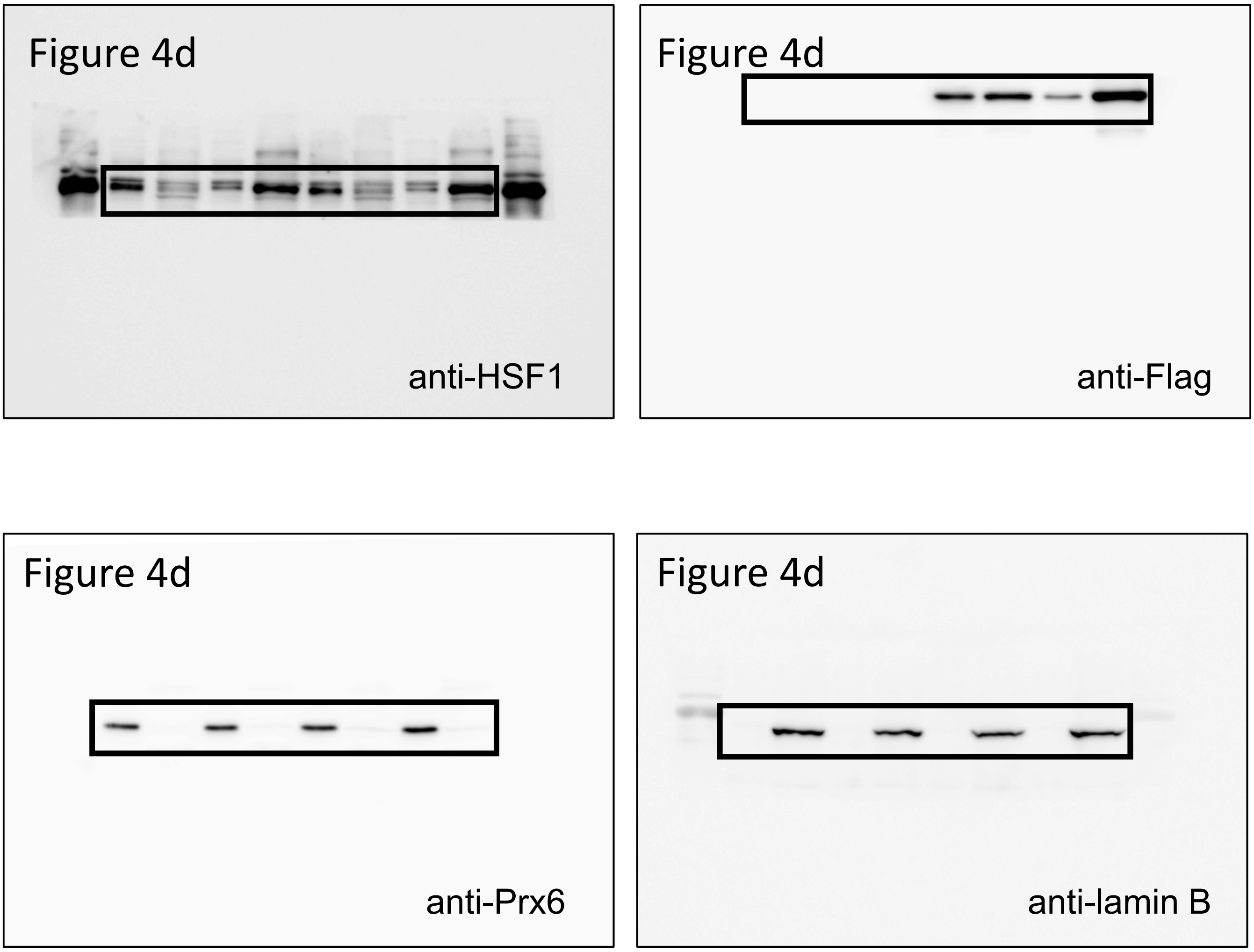

Supplementary Figure 8. Full size gels used in Figure 1-4.

S Figure 9

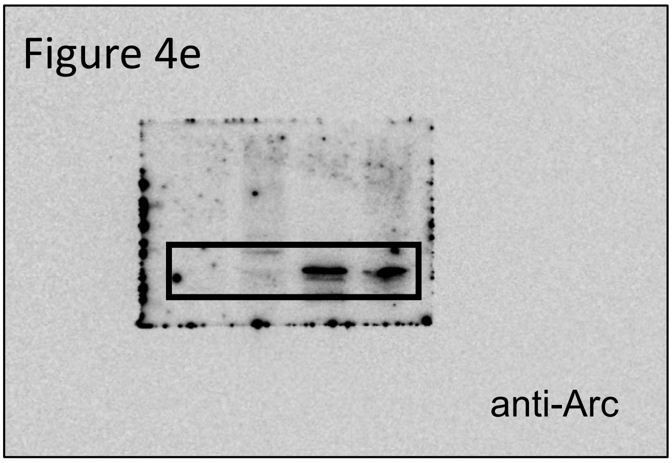

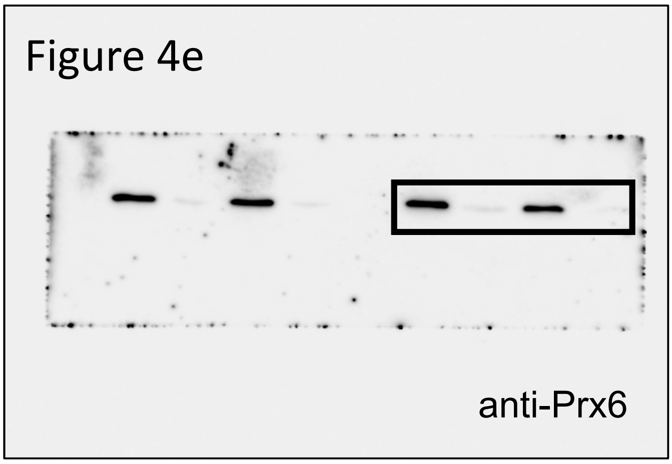

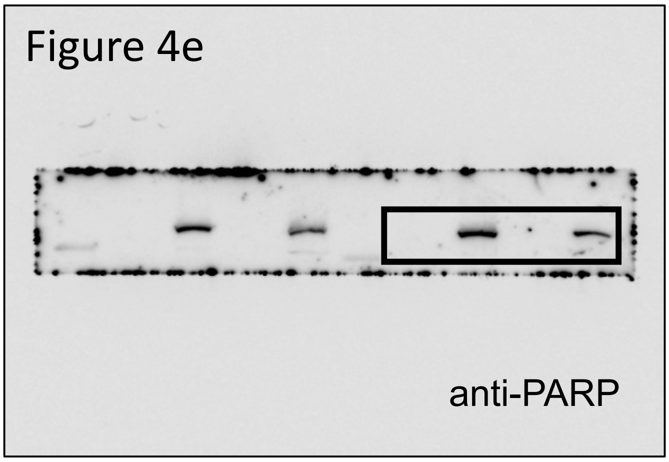

Supplementary Figure 8. Full size gels used in Figure 1-4.
